# Impact of integrated community-facility interventions model on neonatal mortality in rural Bangladesh- a quasi-experimental study

**DOI:** 10.1371/journal.pone.0274836

**Published:** 2023-04-12

**Authors:** Tanvir M. Huda, Suman Kanti Chowdhury, Jatan Bhowmick, Sabrina Sharmin Priyanka, Mohammad Sohel Shomik, Qazi Sadeq-ur Rahman, Mizanur Rahman, Ishtiaq Mannan, Shams El Arifeen

**Affiliations:** 1 Sydney School of Public Health, University of Sydney, Camperdown, Australia; 2 International Centre for Diarrhoeal Disease Research, Bangladesh (icddr,b), Dhaka, Bangladesh; 3 Save the Children, Dhaka, Bangladesh; 4 Carolina Population Center, The University of North Carolina at Chapel Hill, Chapel Hill, North Carolina, United States of America; 5 JSI Research and Training Institute Inc., Denver, Colorado, United States of America; World Health Organization, SWITZERLAND

## Abstract

**Background:**

Neonatal mortality remains unacceptably high in many countries. WHO recommends that all newborns be assessed during the postnatal period and should seek prompt medical care if there is any danger sign. However, in many developing countries, only a small proportion of women receive postnatal care. Also, the quality of care in public health facilities is sub-optimal.

**Methods:**

We designed an intervention package that included community health worker-assisted pregnancy and birth surveillance, post-natal visits to assess newborns on the first, third, seventh and twenty-eighth days of birth, referral for facility-based care, and establishing a newborn stabilization unit at the first level referral health facility. We did a quasi-experimental, propensity-score matched, controlled study in the Sylhet region of Bangladesh. We used a cross-sectional survey method at baseline and endline to measure the effect of our intervention. We considered two indicators for the primary outcome–(a) all-cause neonatal mortality rate and (b) case fatality of severe illness. Secondary outcomes were the proportion of neonates with signs and symptoms of severe illness who sought care in a hospital or a medically qualified provider.

**Results:**

Our sample size was 9,940 live births (4,257 at baseline, 5,683 at end line)**.** Our intervention was significantly associated with a 39% reduction (aRR = 0.61, 95% CI: 0.40–0.93; p = 0.046) in the risk of neonatal mortality and 45% reduction (aRR = 0.55, 95% CI: 0.35–0.86; p = 0.001) in the risk of case fatality of severe illness among newborns in rural Bangladesh. The intervention significantly increased the care-seeking for severe illness at the first-level referral facility (DID 36.6%; 95% CI % 27.98 to 45.22; p<0.001).

**Interpretation:**

Our integrated community-facility interventions model resulted in early identification of severely sick neonates, early care seeking and improved treatment. The interventions led to a significant reduction in all-cause neonatal mortality and case fatality from severe illness.

## Introduction

Neonatal mortality remains unacceptably high. In 2017, 2.5 million neonates died, comprising 202 million disability-adjusted life years [1]. Most deaths were concentrated in sub-Saharan Africa and South Asia [1]. There has only been a modest decline in neonatal mortality over the last decade, which has vastly contributed to the non-attainment of Millennium Development Goal 4 [2]. Based on current rates, it is projected that between 2018 and 2030, 27.8 million children will die within the first 28 days of life [1].

In developing countries, millions of births occur annually without assistance from a skilled birth attendant [3]. Many newborns die from intrapartum-related events, preterm birth complications, sepsis, meningitis, pneumonia, tetanus and diarrhea [2]. Almost seventy percent of neonatal deaths occur during the first seven days of life, and about 1 million neonates die within 24 hours of birth [4]. There are multiple reasons, including the inability of caregivers to identify danger signs, lack of willingness to admit a neonate to hospital, paucity of information about the availability of health services, inadequate referral systems, the inadequacy of transportation facilities; weak linkages of health facilities with communities and poor quality of care at health facilities [3,5,6].

World Health Organization recommends that all newborns be assessed for signs of health problems during the postnatal period and should seek prompt medical care if there is any danger sign. However, in Bangladesh and many other developing countries, only a small proportion of women receive postnatal care from a medically trained provider [7–10]. Studies show that an integrated approach, including community-based care as a vital component, can significantly improve maternal, newborn, and child health outcomes [3]. Community health workers (CHWs) can be crucial in identifying and referring sick newborns for urgent care [11–13]. However, to reduce neonatal mortality and morbidity, the health system needs to be strengthened, and an effective link between healthcare facilities and community-based newborn care is hugely critical [14,15].

In most developing countries, the quality of care in public health facilities is sub-optimal. When sick newborns are hospitalized, the care is often provided too late or ineffective [16]. Fatality rates for sepsis among hospitalized babies are often as high as 45% and are linked to delays in seeking care and poor quality care [17,18]. A study in Bangladesh reported that in most healthcare facilities, the practices to prevent infections were poor, the supplemental oxygen use was inappropriate, and parenteral antibiotics for neonatal infections were inadequate [19,20]. Upazilla health complex (UHC), the first-line referral hospital at the primary level, had no separate room for managing sick newborns. Most UHC had inadequate thermal protection, poor hygiene practices and no systems for prioritizing seriously ill neonates [19]. Strong evidence is that strengthening facility care reduces newborn mortality [21,22].

We designed and evaluated a community and facility-based intervention model interlinked by an active and responsive referral system. Our intervention included community health worker-assisted pregnancy and birth surveillance, post-natal visits to assess newborns on the first, third, seventh and twenty-eighth days of birth, referral for facility-based care and establishing a newborn stabilization unit at the first level referral health facility. We hypothesized that our integrated community-facility interventions model would improve early identification of severe illness in newborns, increase early care seeking and improve the quality of curative care with ultimate improvement in newborn survival.

## Materials and methods

### Study area and population

**Fig 1:**
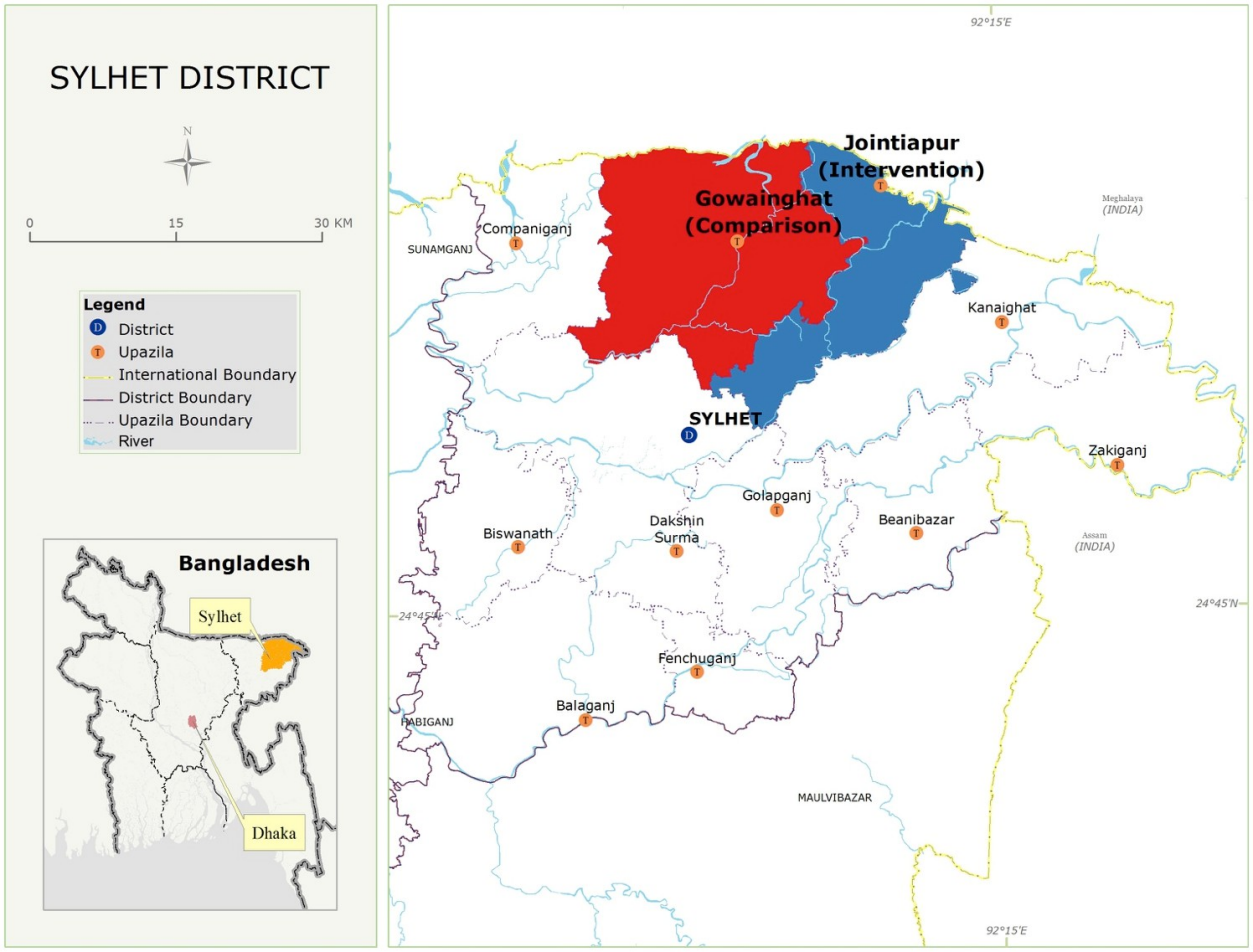
Study area.

Save the Children, Bangladesh implemented the intervention in their “MaMoni”—Maternal and Newborn Care Strengthening Project setting. The Mamoni project was phased out before the start of our study. “Mamoni” was a USAID-funded project aimed at strengthening Maternal and Newborn Care in 10 districts of Bangladesh [23]. The project catalyzed effective scale-up of proven MNC interventions in 10 priority districts and reached a population of approximately 22 million. The project strived to improve equitable access to quality MNC services, especially for the poor and marginalized, for whom the risk of dying is greatest. The study area was Sylhet, which had one of Bangladesh’s highest neonatal mortality rates [24]. The Sylhet district is 200 kilometres north of Dhaka and has thirteen sub-districts with a population of around 4 million. The study area consisted of nine unions from 2 subdistricts of Jaintapur and Gowainghat, with a subpopulation of 200,000 (Fig 1). Union is the lowest administrative unit in Bangladesh, with an average population of 30,000.

### Study design

We conducted a prospective quasi-experimental study. The intervention package included improving the quality of care at the first referral health facility; therefore, an individual or cluster-randomized design was not feasible. The comparison arm received the usual health services government and non-government providers provided. We used a cross-sectional survey method at baseline and endline to measure the effect of our intervention. Propensity Score Kernel Matching with the difference in differences (DID) analysis was applied. The International Centre for Diarrhoeal Disease Research, Bangladesh, ethics review committee approved the trial. We obtained community assent from the village representatives; all women who participated in the study gave verbal consent.

### Intervention

In the intervention arm, the MaMoni program recruited one female CHW for every four villages (about 4000 population) and one trained paramedic (community-based) for every 12 villages (about 12,000 population). Both CHWs and paramedics received six weeks of supervised training on the clinical assessment of neonates. CHWs identified pregnancies through routine household visits every two months and followed women until delivery. A birth notification system was established. A community volunteer, often a traditional birth attendant or active village resident, was responsible for 200–250 households. The volunteer immediately informed CHW about new births through mobile communication. Postnatal visits from the CHW included four contacts (days 0, 3, 7, and 28) with the mother and newborn at home, with the first visit within 24 hours.

During each postnatal visit, CHW recorded symptoms and signs of illness, classified the illness, and determined whether the newborn needed a referral to the hospital. Danger signs were 1) history of difficulty feeding, 2) movement only when stimulated, 3) temperature below 35.5°C, 4) temperature above 37.5°C, 5) respiratory rate over 60 breaths per minute, 6) severe chest in drawings, and, 7) history of convulsions. These signs predict the need for hospitalization with high sensitivity (85%) and specificity (75%) [25]. Once CHW identified a danger sign, the paramedic in the community was immediately informed and made a reassessment. Upon confirmation, the newborn was referred to the stabilization unit at UHC.

Within the intervention area, the quality of newborn care was improved at UHC by establishing a 6-bed specialized newborn stabilization unit (NSU). The project deployed four medical officers and five nurses to run the NSU since the government didn’t have sanctioned posts for the positions needed to run the NSU 24/7 effectively. All doctors and nurses in NSU had training on emergency triage assessment, treatment and sick newborn care from government-designated training institutes. NSU was designed to provide initial care, stabilization of sick newborns, thermal care, resuscitations, and care of low birth weight, not requiring intensive care. Neonatologists from Sylhet Osmany Medical College Hospital, a tertiary level teaching hospital in the Sylhet district, made periodic visits to provide technical and quality assurance. Besides, they were made available to provide emergency advice over the telephone. The consultant Neonatologist was given an honorarium by the project when they visited the special care unit at the intervention area. Referral mechanisms and transportation services with local auto-rickshaw (a motorized, three-wheeled rickshaw for public hire) owners were established to ensure timely referral of severely sick newborns. Drivers resided in the same locality and were available 24/7. Once the health worker referred a case and family members consented to have the newborn treated in the referral hospital, the study’s monitoring officer was contacted, who made all necessary arrangements to transfer the child to the NSU. Upon receiving the information of referral from CHW, the monitoring officer contacted the available auto-rickshaw drivers. He then went to the pre-defined pick-up point with the auto-rickshaw as soon as possible and facilitated the transport of the newborn to the NSU. The monitoring officer also informed the medical officers of the NSU with details of the referred child.

#### Comparison

Women in the comparison arm received the usual health services the government and other non-government health care providers provided. There was no newborn care unit to provide sick newborn care at the Upazilla Health Complex of the comparison Upazilla. The government-employed CHWs were present in both the intervention and comparison arm but were not involved in the management of sick newborns, and neither they had any training in the clinical assessment of neonates.

### Data collection

Two cross-sectional surveys were conducted at baseline between May 2013 and July 2013 and at the end of February 2015 and May 2015. All women who delivered in the last 12 months from the survey date were included in the survey. Based on the annual crude birth rate of 25 per 1000 population, we anticipated getting a minimum of 2000 live births in each arm at baseline and end line. We estimated this sample size would be adequate to show a 40% effect size for neonatal (primary outcome) with more than 80% power and a 5% significance level (two-sided test). We had a total number of 9,940 live births (4,257 at baseline, 5,683 at end line). Among them, we had 119 multiple outcomes in the intervention arms (2.2%) and 102 multiple outcomes in the comparison arm (2.0%). Our surveys had a low participant refusal rate (less than 1%). Two groups of interviewers from an independent research firm—Associates for Community and Population Research, collected the data. The first group was responsible for household listing and identifying eligible women who had a pregnancy outcome within the last 12 months. The second group interviewed the mother of the eligible child. Each group consisted of 4 female enumerators and one supervisor who all spoke local languages. The interviewers were selected meticulously, followed by adequate training and field practices to ensure the quality of data collection. Survey responses were checked for consistency and accuracy by supervisors in the field. A separate quality control team with one senior staff and six quality control officers oversaw data collection. Interviewers collected data on pregnancy, delivery, newborn care practices, survival status of the index child plus socio-demographic variables, reproductive health characteristics, maternal health service utilization, newborn care and care-seeking, expenditure related to neonatal illness and maternal health knowledge. The information was collected in a structured format of the questionnaire, all retrospective. Whether or not a newborn had a severe illness during the 28 days of their life, including the type of treatment sought, was decided based on answers to a series of illness-related questions in the structured questionnaire.

### Outcomes

We considered two indicators of the primary outcome–(a) all-cause neonatal mortality rate (per 1000 live births), which included all deaths in the first 28 days of life (b) case fatality of severe illness that is the proportion of neonate deaths from among those who had signs and symptoms of severe illness. We defined severe illness using newborn danger signs reported in The Young Infants Clinical Signs Study Group [25] and the Bangladesh Neonatal Health Strategy [26]. We asked each respondent a series of questions regarding the signs and symptoms of their newborn during the first 28 days. We identified severe illness in newborns if their mother reported the presence of any 1 of the following signs and symptoms: unusually cold/clammy skin, high body temperature, unconscious/no movement or lethargic, caregivers report of convulsions, rapid breathing or difficulty in breathing, unable to breastfeed or severe chest indrawing. This definition of severe illness is recommended for studies in low-resource settings [27,28]. We present severe illness as a binary outcome variable indicating the presence or absence of the outcome of interest.

Secondary outcomes included the proportion of neonates with signs and symptoms of severe illness seeking care. The type of treatment received was categorized into four types: (1) in a hospital (government or private), (2) by a medically trained provider (with an MBSS degree) outside of a hospital setting; (3) by a CHW or paramedic; (3) by other providers, including homoeopathic doctors, village doctors, pharmacists, etc. We assigned the treatment type hierarchically, which means if a newborn was taken to a hospital, he was included in that category, even if initially treated by CHWs or paramedics. The coverage of intervention was assessed by the mean number of CHW visits after birth, the proportion of neonates visited by a CHW within 24 hours, the proportion of neonates referred to a health facility by a CHW and the proportion of neonates that complied with the referral.

### Statistical analysis

We tested whether the intervention affected our outcomes using difference-in-differences analysis with propensity score kernel matching. The difference-in-difference (DID) analysis compared changes in outcomes over time between intervention and comparison arms. In our study, the difference in case fatality of severe illness, neonatal mortality and care-seeking for severe illness in the treatment arm before and after the intervention, minus the corresponding change in the comparison arm, provided an estimate of the impacts of our intervention. Ideally, the intervention and comparison arms would have similar characteristics to allow valid comparisons. However, in the absence of randomization, treated and control subjects are not comparable before treatment.

We used propensity scores to create a comparison arm with covariates balanced with the intervention arm. The covariates used to create the propensity score were maternal age, maternal education, maternal health services (four or more antenatal care visits), postnatal care visits within 48 hours, parity, Sex of child, and birth outcome (singleton or multiple). We used the Stata "diff" package with a kernel matching option. In Kernel-based propensity score matching, comparison subjects are weighted by their distance in propensity score from intervention subjects. Thus, the more "similar" the comparison subjects were to intervention subjects, the more weight they were given. The advantage is that kernel matching uses weighted averages of comparison arm participants to maximize precision (lower variance) without worsening bias (giving greater weight to better matches).

Relative Risks and 95% CIs for case-fatality and neonatal mortality rates were calculated using modified Poisson regression models. We controlled for maternal age, maternal education, maternal health services (four or more antenatal care visits), postnatal care visits within 48 hours, parity, Sex of the child, and birth outcome (singleton or multiple) for the primary outcomes. For our regression analysis, we also included an interaction term between the survey’s timing and intervention to adjust for any difference in the mortality at the baseline. Data were analyzed using STATA version 16 (StataCorp, TX, USA).

## Results

The mean age of mothers, the proportion of male and female children and the mean household size were balanced between arms at baseline and end line. Mean years of education and percentage of mothers with a first child were slightly higher in the intervention arm at baseline and end line. The intervention arm had higher socioeconomic conditions than the comparison arm (Table 1).

**Table 1:** Demographic, maternal, neonatal and household characteristics.

	Comparison		Intervention	
	Baseline (n = 2,066)	End line (n = 2,867)	Baseline (n = 2,191)	End line (n = 2,816)
Mean maternal age in years (SD)	24.8(5.8)	24.3(5.6)	24.6(5.9)	24.1(5.6)
Mean years of maternal education (SD)	3.1(3.2)	3.6(3.4)	4.2 (3.3)	4.7(3.3)
Birth order				
First child	21.6%	23.1%	27.8%	29.3%
Second or third child	38.9%	39.3%	39.5%	42.6%
Fourth or higher	39.5%	37.6%	32.6%	28.1%
Singleton or multiple births				
Singleton	97.7%	98.5%	97.7%	97.6%
multiple	2.3%	1.5%	2.3%	2.3%
Sex				
Male	52.4%	51.2%	50.7%	50.8%
Female	47.6%	48.8%	49.3%	49.2%
At least 4 Antenatal care visits				
Yes	1.9%	2.7%	5.6%	6.3%
No	98.1%	97.3%	94.4%	93.7%
Post Natal Care within 48 hours				
Yes	6.6%	9.2%	18.1%	22.8%
No	93.4%	90.8%	81.9%	77.2%
Household size	7.2(3.3)	7.4(3.6)	7.1(3.4)	6.9(3.4)
Household wealth quintile				
1st (poorest)	23.8%	24.2%	14.0%	13.5%
2nd	22.6%	21.8%	16.5%	18.4%
3rd	19.5%	17.7%	21.4%	21.2%
4th	18.2%	19.8%	23.4%	21.6%
5th (wealthiest)	15.6%	16.2%	24.5%	25.1%

Data are n (%) or mean (SD).

Study CHW was able to visit 88% of newborns in the intervention arm. Of the visits, 33% were made within the first 24 hours, and 78% were made within 72 hours, with 12% of all newborns referred to a facility. Of these, 88% complied with the referral (Table 2).

**Table 2:** Intervention coverage.

Number (%) of the child to whom CHW made a visit	**2946 (88.4%)**
Mean (SD) number of visits by CHW after birth	2.6 (1.2%)
Number (%) of the child to whom CHW visited within 24 hours	822 (33.0%)
Number (%) of the child to whom CHW visited within 72 hours	1936 (77.7%)
Number (%) of the child that was referred to a health facility	314 (12.6%)
Number (%) of the child that complied with the referral	277 (88.2%)

Data are n (%).

The incidence rates of severe illness increased from 71 per 1000 live births at baseline to 103 per 1000 live births at the intervention arm’s end line. The incidence of severe illness in the comparison arm increased from 68 at the baseline to 100 per 1000 live births at the end line (Table 3).

**Table 3:** Incidence rates of severe illness (95% confidence interval) per 100 live births.

	No of cases	At-risk	Rate per 1000 live births	95%CI	
Comparison					
Baseline	141	2066	68.0	58.0	79.0
End line	288	2867	100.0	8.9	112.0
Intervention					
Baseline	156	2191	71.0	61.0	82.0
End line	292	2816	103.0	92.0	115.0

Our community-facility interventions model significantly reduced neonatal mortality in the intervention upazilla compared with control upazilla (DID -15.0%; 95% CI: -28.7 to -12.8; p<0.046). The intervention had larger impact on the case fatality of severe illness (DID: -19.9%; 95% CI: -20.02 to -19.7; p 0.001).

Our intervention resulted in a large increase in care seeking from the first-level referral facility (DID 36.6%; 95%CI 27.98 to 45.22; p<0.001). There was a small non-significant increase in care seeking from the medical college hospital (DID 1.8; 95%CI -6.628 to 10.23; p = 0.678) and medically qualified providers outside the hospital settings (DID 1.6; 95%CI -8.984 to 12.18; p = 0.761). Care-seeking from private hospitals (DID—3.0; 95%CI -9.076 to 3.076; p = 0.342) and informal providers (DID– 21.3; 95%CI -33.26 to -9.344; p<0.001) decreased in the intervention arm (Table 4).

**Table 4:** Mortality outcome and percentage of care-seeking for newborn illness.

	Baseline				Endline					
	Intervention	Comparison	Difference	p-value	Intervention	Comparison	Difference	p-value	Difference-in-differences	p-value
**Mortality outcome** ^1^										
Neonatal mortality rate (per 1000 live births)	37.0	32.0	5.0	0.355	31.0	40.0	-10.0	0.049	-15.0	0.046
Case fatality rate of severe illness (%)	39.7	27.2	12.5	0.012	17.8	25.3	-7.5	0.039	-19.9	0.001
**Care seeking for severe illness**										
Treated in Upazilla Health Complex (%)	5.7	5.5	0.2	0.959	43.5	6.7	36.8	<0.001	36.6	<0.001
Treated in Medical College (%)	10.5	8.7	1.8	0.554	15.4	11.8	3.6	0.236	1.8	0.678
Treated in a Private clinic (%)	7.0	4.4	2.6	0.246	5.5	5.9	-0.4	0.857	-3.0	0.342
Treated by a medically qualified provider outside hospital settings (%)	16.5	17.2	0.7	0.851	22.7	23.6	0.9	0.808	1.6	<0.761
Treated by an informal provider (%)	49.7	41.5	8.2	0.058	13.7	26.9	-13.2	0.002	-21.3	<0.001

^1^Adjusted for maternal age, maternal education, four or more ANC visit, PNC visits within 48 hours, parity, Sex of child, birth outcome (singleton or multiple), household size, household wealth and Kernel RCS.

^2^Informal Providers included Homeo doctors, Ayurvedic doctors, Kobiraj, Pharmacy shop keeper and other village doctors.

After adjusting for maternal, neonatal, and household factors, the intervention reduced the risk of neonatal death by 39% (ARR 0.61; 95%CI 0.40–0.93; p = 0.021) and of case fatality by 45% (ARR 0.55; 95%CI 0.36–0.86; p = 0.009). The likelihood of seeking newborn curative care in the first-level referral facility was almost seven times more in the intervention arm (ARR 6.95; 95%CI 1.82–2.65; p<0.001). The likelihood of seeking care from a private clinic (ARR 0.42; 95%CI 0.21–0.85, p = 0.017) and from an informal provider (ARR 0.34; 95%CI 0.25–0.47, p<0.001) was significantly lower in the intervention arm (Table 5).

**Table 5:** Effect of intervention on neonatal mortality, case fatality of severe illness and care-seeking for severe illness.

	Adjusted Risk Ratio	95%CI	p-value
**Mortality outcome** ^1^			
Neonatal mortality	0.61	0.40–0.93	0.021
Case fatality rate	0.55	0.35–0.86	0.009
**Care seeking for severe illness**			
Treated in UHC	6.95	4.21–11.47	<0.001
Treated in Medical College	1.04	0.67–1.62	0.849
Treated in private/NGO clinic	0.42	0.21–0.85	0.017
Treated by a medically qualified provider	1.38	0.98–1.95	0.064
Treated by other informal providers	0.34	0.25–0.47	<0.001

^1^Adjusted for maternal age, maternal education, four or more ANC visit, PNC visits within 48 hours, parity, Sex of child, birth outcome (singleton or multiple), household size, and household wealth. An interaction term between the timing of the survey and intervention was included to adjust for any difference in the mortality at the baseline.

## Discussion

Our combination of community and facility-based interventions interlinked by an active and responsive referral system helped early identification of severely ill newborns, facilitated quick and effective referral, and ensured the quality of newborn care at the first-level referral health facility. This led to a substantial increase in hospital care-seeking and a significant reduction in case fatality from severe illness and neonatal mortality from all causes. Overall, the intervention reduced the risk of neonatal death by more than one-third—similarly, case fatality risk due to severe illness was reduced by almost half. The Effect of community-based intervention packages in improving neonatal health outcomes and delivered through CHWs and facility-based services was found to be encouraging in other studies [3,29–31].

Our study had some limitations. Because an individual or cluster randomized control trial was not feasible, we used a quasi-experimental design. However, we used DID analysis with propensity score matching to reduce bias due to baseline imbalance or potential confounders. Another limitation was that we relied on recalled data about newborn danger signs, newborn-care practices, and mortality. We used standardized data collection methods; therefore, recall biases were expected to be similar across both arms. We used a similar case definition for severe illness as in other settings [32,33].

We have shown that CHWs could identify severely ill newborns and motivate caregivers to seek hospital care. CHWs visited a newborn four times during the first 28 days. A significant increase in care-seeking from the first-level referral facility in the intervention arm suggests that the CHW home visits strategy was highly effective. Previous studies indicate that community strategies of CHW home visits and community mobilization effectively increase health facility utilization [34–37]. Studies have also shown that when CHWs are appropriately trained, supported and supervised, they can correctly identify newborn illnesses and motivate caregivers to seek health care [34–36,38].

A well-functioning system through which a sick newborn is referred to a facility to obtain appropriate treatment is essential to reduce neonatal mortality [39,40]. Timely care-seeking can avert neonatal deaths by 30–60% [40,41], but access and transport costs are barriers to referral compliance in many low-resource areas in developing countries [42,43]. Lack of coordination between community workers and referral facilities impedes referral compliance [44]. A referral mechanism and 24/7 transportation service were established in our study to ensure timely referral of severely sick newborns. The monitoring officer would also inform the duty doctor of the referral facility with details of patients. Our study achieved a very high compliance rate with referrals, with 88% of referred cases seeking care at the designated health care facility. Much of this success can be attributed to the established transport mechanism. Previous studies suggest timely referral and referral compliance are critical to increasing newborn survival [39–41,45].

Our study also concerted efforts to improve the quality of newborn care at the first-level referral facility. Quality of care is increasingly recognized as one of the most critical components for newborn survival. It is well documented that high coverage alone is not enough to reduce newborn mortality. To improve neonatal survival substantially, it is crucial to enhance the quality throughout the continuum of care [46]. In Bangladesh, first-level referral health facilities lack adequate health workers, essential medicines, and supplies and thus struggle to provide appropriate care to newborns [47]. As a result, severe newborn illness’s case fatality is often high in such facilities [48]. One of our intervention’s major components was improving care at the sub-district level by establishing a functional newborn stabilization unit. This achieved a significant decline in case fatality from newborn illness. This is consistent with the results from other studies in a similar context [21,22,49].

We found the comparison arm’s neonatal mortality rate had around a 16% increase at the end line than baseline estimation. The comparison area’s baseline neonatal mortality rate was much lower than the regional mortality rate reported in the national household survey [24]. The lower neonatal mortality rate at the baseline in the comparison arm could be attributed to two large maternal and child health programs (ACCESS and MaMoni) implemented in the comparison area before the start of our study. However, as the programs were phased out from the comparison area, their effects faded, and at the endline survey, we found a mortality rate similar to the regional average [24].

Between the 1990s and 2017, neonatal mortality in Bangladesh fell by 42%. This annual reduction rate was lower than the regional and global averages of around 2.0% and 2.6% per year, respectively [1]. More innovative intervention packages are needed to reduce neonatal mortality further. Infections, including pneumonia, are responsible for one-third of deaths during the first 28 days. Effective management of neonatal infections can be undertaken through community-based strategies [50]. More attention is needed to establish effective referral mechanisms and improve newborn care quality at first-level referral health facilities. Investments should be made to develop specialized newborn care units at subdistrict and district health facilities. Our study did not include any maternal component in the intervention package. Maternal care, mostly adequate nutrition during pregnancy, the recommended number of antenatal and postnatal care visits, skilled attendance at birth, and immediate treatment of any maternal complication, are incredibly critical. Any intervention package to improve neonatal survival should combine maternal and neonatal care elements to achieve optimal impact.

One of our study’s major strengths was that the interventions’ delivery was designed around the existing public health structure. Hence the interventions can likely be feasible and sustainable through local health systems. The government has more than 40,000 CHWs (Health Assistants and Family Welfare Assistants) responsible for providing reproductive, maternal, newborn, and child health services at the doorstep and in outreach centres. These CHWs can be given an orientation and responsibility for assessing newborns. Referrals can be managed by functioning community support groups facilitated by the government’s community clinic initiative. Strengthening hospital care, particularly for sick newborns, is already a government plan to achieve the Sustainable Development Goal.

## Supporting information

S1 File (DOCX)

S2 File (PDF)

S3 File (PDF)
