# Supplementary material for: Impact of integrated community-facility interventions model on neonatal mortality in rural Bangladesh- a quasi-experimental study
Source: PLoS One. 2023 Apr 12;18(4):e0274836. doi: 10.1371/journal.pone.0274836 (PMC10096467; doi:10.1371/journal.pone.0274836)
Supplement: S3 File — (PDF) [file pone.0274836.s003.pdf]

ASSESSING THE EFFECTIVENESS OF TARGETED APPROACH FOR NEONATAL HEALTH AND FAMILY PLANNING (FP) SERVICES IN RURAL BANGLADESH

Questionnaire for
Recently Delivered Women (RDW)
SYLHET

Associates for Community and Population Research (ACPR)
3/10, Block A, Lalmatia, Dhaka-1207, Bangladesh

সনাক্তকরণ IDENTIFICATION

	নাম Name	কোড Code	
জেলা	Sylhet	☐☐	সাক্ষাৎকার শুরু হবার সময়:
উপজেলা		☐☐	
ইউনিয়ন		☐☐☐	|_____|_____| : |_____|_____| ঘন্টা মিনিট
ক্লাস্টার নং		☐☐☐☐	
গ্রাম		☐☐☐☐	
বাড়ির নাম ও নং		☐☐☐	
খানা নং		☐☐	সাক্ষাৎকার শেষ করার সময়:
সম্প্রতি প্রসবকারী মহিলার নাম এবং লাইন নম্বর		☐	
সম্প্রতি প্রসবকারী মহিলার স্বামীর নাম			|_____|_____| : |_____|_____| ঘন্টা মিনিট
খানা প্রধানের নাম			
সম্প্রতি প্রসবকারী মহিলা/ স্বামীর Mobile নম্বর	|__|__|__|__|__|__|__|__|__|__|__|		

INTERVIEWER'S VISIT AND STATUS

	পরিদর্শন-১	পরিদর্শন-২	পরিদর্শন-৩	শেষ পরিদর্শন Final Visit	
তারিখ	|_____|_____|-|_____|_____|-|_____|_____|	|_____|_____|-|_____|_____|-|_____|_____|	|_____|_____|-|_____|_____|-|_____|_____|	তারিখ	|_____|_____|-|_____|_____|-|_____|_____|
সাক্ষাৎকারগ্রহনকারীর নাম				সাক্ষাৎকারগ্রহনকারীর কোড	|_____|_____|_____|
**Result code***	|_____|_____|	|_____|_____|	|_____|_____|	**Result code**	|_____|_____|
পরবর্তী পরিদর্শন	**তারিখঃ**	**তারিখঃ**	**তারিখঃ**	মোট পরিদর্শন	|_____|
	**সময়ঃ**	**সময়ঃ**	**সময়ঃ**		

RESULT CODES*:

01. ইন্টারভিউ সমাপ্ত
02. বাড়ি পরিদর্শনের সময় খানার কোন সদস্যকে বা উপযুক্ত কাউকে পাওয়া যায় নাই
03. ইন্টারভিউ বাতিল
04. ইন্টারভিউ দিতে রাজী নয়
05. বাসস্থানটি খুঁজে পাওয়া যায় নাই
06. সম্প্রতি প্রসবকারী মহিলা অনুপস্থিত
07. গত ১৫ মাস সময়ের (০১ **নভেম্বর** ২০১১ থেকে ৩১ জানুয়ারী ২০১৩) মধ্যে এই খানার কোন মহিলার গর্ভ শেষ হয় নাই
08. অন্যান্য ______________________________ (উলে- খ করুন)

তত্ত্বাবধায়ন	নাম	কোড	তারিখ
Reviewed by Supervisor		|____|____|____|	|___||___|-|___||___|-|___||___|
Checked by Field Editor		|____|____|____|	|___||___|-|___||___|-|___||___|
Reviewed by Office Editor		|____|____|____|	|___||___|-|___||___|-|___||___|
Keyed by		|____|____|____|	|___||___|-|___||___|-|___||___|

আন্তর্জাতিক উদারাময় গবেষণা কেন্দ্র, বাংলাদেশ (ICDDR,B)

সাক্ষাৎকারে অংশগ্রহণকারীর মৌখিক সম্মতি আদায়ের জন্য তথ্যপত্র

Protocol Title: Assessing the effectiveness of targeted approach for neonatal health and family planning (FP) services in rural Bangladesh

Investigator's name: Dr. Tanvir Mahmudul Huda
Organization: International Centre for Diarrhoeal Diseases Research, Bangladesh (icddr,b)

গবেষণার উদ্দেশ্য

বাংলাদেশে এখনও নবজাতকের মৃত্যু এবং নারীর প্রজনন হার অনেক বেশী। নবজাতক ও নারীর প্রজনন স্বাস্থ্যের আরও উন্নতির লক্ষ্যে আইসিডিডিআর, বি, সেইভ দ্যা চিল্ড্রেন এবং এনজেন্ডারহেল্থ একটি গবেষণা পরিচালনা করছে। এই গবেষণার প্রধান উদ্দেশ্য হল নবজাতকের মৃত্যু ও অধিক প্রজননের জন্য সবচেয়ে ঝুঁকিপূর্ণ মা ও বিবাহিত মহিলাদের উপর বিশেষ জোর দিয়ে একটি কার্যক্রম পরিচালনা করা এবং তার যথার্থতা যাচাই করা। আপনার নিকট হতে প্রাপ্ত তথ্য আমাদেরকে নবজাতক ও প্রজনন স্বাস্থ্য সেবা উন্নয়নের জন্য সাহায্য করবে।

আপনাকে কেন নির্বাচিত করা হলো

যেহেতু আপনি একজন সদ্য প্রসূতি মা হওয়ায়, আমরা আপনাকে এই গবেষণায় অংশগ্রহণ করার জন্য আমন্ত্রণ জানাচ্ছি ।

পদ্ধতি এবং কার্যপ্রণালী

আপনি এই গবেষণায় অংশগ্রহণে রাজী থাকলে আমরা আপনার খানা, আর্থ-সামাজিক অবস্থা, শিক্ষা, গর্ভ ও জন্মকালীন ইতিহাস, আপনার শেষ গর্ভকালীন, প্রসব কালীন ও প্রসব-পরবর্তী ইতিহাস এবং নবজাতকের পরিচর্যা ও জন্ম মৃত্যুর ইতিহাস এবং পরিবার পরিকল্পনা সম্পর্কে তথ্য সংগ্রহ করব। এই সাক্ষাৎকার গ্রহণের জন্য হয়ত ৪৫-৬০ মিনিটের মত সময় লাগতে পারে।

ঝুঁকি এবং সুবিধা

এই গবেষণায় অংশগ্রহণের জন্য আপনার ঝুঁকির সম্ভাবনা খুবই কম। আমরা শুধু গবেষণা কার্যক্রমের অংশ হিসাবে আপনার কাছ থেকে উপরে উলে- খিত তথ্য সংগ্রহ করব যার জন্য আপনাকে কিছু সময় ব্যয় করতে হবে ।

এই গবেষণায় অংশগ্রহণের জন্য আপনি সরাসরি উপকার পাবেন না। তবে আপনার কাছ থেকে আমরা যে তথ্য পাব তা মাঠ পর্যায়ে মা এবং শিশুদের স্বাস্থ্যের উন্নয়নে নীতিমালা তৈরী ও কার্যক্রমের দিক নির্দেশনা দিবে যা পরবর্তীতে বাংলাদেশ ও অন্যান্য স্থানে শিশু স্বাস্থ্য কার্যক্রম উন্নয়নে কাজে লাগবে।

গোপনীয়তা এবং বিশ্বস্ততা

আপনি যে তথ্য দিবেন সেগুলো সম্পূর্ণ গোপন রাখা হবে এবং তা তালাচাবি দিয়ে আটকানো থাকবে। গবেষণার গবেষকরা, সম্ভাব্য ক্ষেত্রে গবেষণার পরীক্ষক, এবং বিশেষ প্রয়োজনে আইনী সংস্থা ছাড়া অন্য কেউই আপনার দেওয়া তথ্য জানতে পারবে না। আপনাকে চিনতে পারার মত সকল তথ্য খুবই সাবধানতার সাথে ব্যবহার করা হবে,্এবং অল্প সংখ্যক ব্যক্তির বাইরের কেউই তা জানতে পারবেন না।

তথ্যের ভবিষ্যৎ ব্যবহার

আপনার দেয়া তথ্যগুলো সরকার ও বেসরকারী সংস্থা কর্তৃক মা, নবজাতক ও শিশু স্বাস্থ্য সেবা প্রদানের কার্যকরী পদ্ধতি উন্নয়নে সাহায্য করবে।

অংশগ্রহণ না করা বা প্রত্যাহার করার অধিকার

এই গবেষণায় আপনার অংশগ্রহণ হবে সম্পূর্ণ স্বতঃস্ফূর্ত এবং এই গবেষণায় আপনি অংশগ্রহণ করবেন কি করবেন না এটা আপনার সম্পূর্ণ নিজের উপর নির্ভর করবে । আপনি যে কোন সময় গবেষণায় অংশগ্রহণ না করার সিদ্ধান্ত নিতে পারেন। আপনি যদি এই কাজে অংশগ্রহণ নাও করেন অথবা আপনি যদি কখনও গবেষণা থেকে নিজেকে সরিয়ে নেন তবুও আপনি ও আপনার পরিবার আইসিডিডিআর,বি অথবা এই এলাকার বিভিন্ন হাসপাতাল/ক্লিনিক/স্বাস্থ্য সেবা কেন্দ্র হতে বরাবর যে স্বাস্থ্য সেবা পেতেন তার কোন পরিবর্তন হবে না।

ক্ষতিপূরণ নীতি

আগেই বলা হয়েছে যে এই কাজে এবং এই গবেষণায় অংশগ্রহণের জন্য আপনাকে কোন প্রকার খরচ দেয়া হবে না। আপনার অংশগ্রহণ সম্পূর্ণ স্বতঃস্ফূর্ত।

আপনার প্রশ্নের উত্তর / যোগাযোগ

এই গবেষণা সম্পর্কে আপনার কোন প্রশ্ন থাকে অথবা আপনি মনে করেন যে অসৎ আচরণ করা হয়েছে অথবা গবেষণায় অংশগ্রহণ করে যদি কোন কিছুতে আপনি কষ্ট পেয়ে থাকেন, তাহলে আপনি ডাঃ তানভীর মাহমুদুল হুদা, প্রজেক্ট কর্ডিনেটর, সিসিএএইচ, আইসিডিডিআর,বি. ঢাকা, বাংলাদেশ, সাথে যে কোন সময় যোগাযোগ করতে পারেন। ফোন: ৮৮১০১১৫ (ঢাকা)। আপনি এম.এ. সালাম খান, আই আর বি সেক্রেটারিয়েট, রিসার্চ এডমিনিস্ট্রেশন, আইসিডিডিআর,বি এর সাথেও যোগাযোগ করতে পারেন। ফোন: ৯৮৮৬৪৯৮ (ঢাকা) ।

আপনি যদি আমাদের প্রস্তাবে এই গবেষণায় অংশগ্রহণের জন্য রাজী হন তাহলে, দয়া করে আপনি নিম্নলিখিত স্থানে স্বাক্ষর প্রদান করুন অথবা আপনার বাম হাতের বুড়ো আঙ্গুলের ছাপ দিন। আপনার সহযোগিতার জন্য অনেক ধন্যবাদ ।

____ _______________

স্বাক্ষর/বাম হাতের বুড়ো আঙ্গুলের ছাপ অংশগ্রহণকারী/অভিভাবক/দেখাশুনার দায়িত্বে যে ছিলেন তারিখ

____________________ ________ ____________________ ________

সাক্ষীর স্বাক্ষর অথবা বাম হাতের বুড়ো আঙ্গুলের ছাপ তারিখ প্রধান গবেষকের/তার পক্ষে স্বাক্ষর তারিখ

(দ্রষ্টব্য: প্রধান গবেষকের প্রতিনিধি তার পুরো নাম ও পদবী লিখে স্বাক্ষর করবেন)

Section A: Respondent's and her Husband's Background

এখন আমি আপনার এবং আপনার স্বামী সম্পর্কে কিছু প্রশ্ন জিজ্ঞেস করতে চাই।

No.	QUESTIONS AND FILTERS	CODING CATEGORIES	SKIP
101.	আপনি কোন্ সালের কোন্ মাসে জন্মগ্রহন করেছিলেন?	মাস..<br>জানি না .... 98<br>সাল..<br>জানি না 9998	
102.	বর্তমানে আপনার বয়স কত?<br>**102 এর সাথে 101 মিলিয়ে দেখুন, অসামঞ্জস্য হলে 102 এবং/বা 101 সংশোধন করুন।**	বৎসর **(পূর্ণ বৎসর)**	
103	আপনি কি কখনও স্কুলে বা মাদ্রাসায় লেখাপড়া করেছেন?	হ্যাঁ, স্কুল .... 1<br>হ্যাঁ, মাদ্রাসা .... 2<br>হ্যাঁ, উভয়ই .... 3<br>না 4	4 → **105**
104.	আপনি সর্বোচ্চ কোন ক্লাস পাশ করেছেন?<br>**কোন ক্লাস পাশ না করলে 00 লিখুন।**	ক্লাস	
	**আপনি হয়ত জানেন যে কোন কোন মহিলা নগদ টাকা বা জিনিসপত্রের বিনিময়ে কাজ করে, কেউ জিনিসপত্র বিক্রি করে, কেউ নিজের ছোট ব্যবসায় বা পারিবারিক খামারে বা ব্যবসায় কাজ করে।**		
105.	বর্তমানে আপনি **নগদ টাকা বা জিনিসপত্রের বিনিময়ে** কোন কাজ করছেন কি?	হ্যাঁ .... 1<br>না 2	2 → **108**
106.	প্রধানতঃ আপনি কি কাজ করেন?<br><br>**একাধিক পেশার সাথে জড়িত হলে প্রধান পেশার নাম নিচে লিখে ডান দিকের কোড বৃত্তায়িত করুন।**<br><br>পেশাঃ________________________________	শারীরিক পরিশ্রম ভিত্তিক কাজঃ<br>নিজের জমিতে চাষাবাদ বা বর্গাচাষী............. 01<br>দিন মজুর/অদক্ষ শ্রমিক (গৃহস্থালী, কৃষিভিত্তিক ইত্যাদি) .... 02<br>দক্ষ শ্রমিক (দীর্ঘ মেয়াদে চুক্তিবদ্ধ/কাঠমিস্ত্রি/ রাজমিস্ত্রি/জেলে) .... 03<br>নৌকাচালক .... 04<br>অ-শারীরিক পরিশ্রম ভিত্তিক কাজঃ<br>নিজস্ব ব্যবসা....................................... 05<br>চাকুরীজীবি/পেশাজীবি (ডাক্তার, প্রকৌশলী, উকিল, শিক্ষক) .... 06<br>অন্যান্য_____________ ....96<br>(নির্দিষ্ট করুন)	
107.	আপনার মাসে আনুমানিক কত টাকা আয় হয়?<br>যদি আয় টাকাতে না হয়ে চাল, গম বা অন্য কিছুতে হয় তবে তার পরিমাণ উল্লেখ করুন।<br>**না হলে 000 লিখুন।**	আয়................. টাকা<br>চাল .... কেজি.<br>গম.................... কেজি<br>অন্যান্য_____________ ....996<br>(নির্দিষ্ট করুন)	

No.	QUESTIONS AND FILTERS	CODING CATEGORIES	SKIP
108	আপনি বর্তমানে বিবাহিতা, বিচ্ছিন্না, পরিত্যক্তা, বিধবা না তালাকপ্রাপ্তা?	বর্তমানে বিবাহিতা ....1<br>বিচ্ছিন্না ....2<br>পরিত্যক্তা ....3<br>তালাকপ্রাপ্তা ....4<br>বিধবা ....5<br>কখনও বিয়ে হয়নি 6	2–5 → 201<br>6 → 1026
109	বর্তমানে আপনার স্বামীর বয়স কত?	বৎসর (পূর্ণ বছরে) .... [ ][ ]<br>জানিনা 97	
110	আপনার স্বামী কখনও স্কুলে বা মাদ্রাসায় লেখাপড়া করেছেন কি?	হ্যাঁ, স্কুল ....1<br>হ্যাঁ, মাদ্রাসা ....2<br>হ্যাঁ, উভয়ই ....3<br>না ....4<br>জানি না 7	4, 7 → 112
111	আপনার স্বামী সর্বোচ্চ কোন ক্লাস পাশ করেছেন?<br>**কোন ক্লাস পাশ না করলে 00 লিখুন।**	ক্লাস .... [ ][ ]<br>জানি না 97	
112	বর্তমানে আপনার স্বামী আয় রোজগারের জন্য কোন কাজ করেন কি?	হ্যাঁ ....1<br>না 2	2 → 201
113	আপনার স্বামীর প্রধান পেশা কি?<br><br>**একাধিক পেশার সাথে জড়িত থাকলে প্রধান পেশার নাম নিচে লিখে ডান দিকের কোড বৃত্তায়িত কর ্ন।**<br><br>পেশাঃ ________________________________	<u>শারীরিক পরিশ্রম ভিত্তিক কাজঃ</u><br>নিজের জমিতে চাষাবাদ বা বর্গাচাষী............01<br>দিন মজুর/অদক্ষ শ্রমিক (গৃহস্থালী,<br>কৃষিভিত্তিক ইত্যাদি) ....02<br>দক্ষ শ্রমিক (দীর্ঘ মেয়াদে চুক্তিবদ্ধ/কাঠমিস্ত্রি/<br>রাজমিস্ত্রি/জেলে) ....03<br>রিকসাচালক/ভ্যানচালক/নৌকাচালক............04<br><u>অ-শারীরিক পরিশ্রম ভিত্তিক কাজঃ</u><br>নিজস্ব ব্যবসা.......................................05<br>চাকুরীজীবি/পেশাজীবি (ডাক্তার,<br>প্রকৌশলী, উকিল, শিক্ষক) ....06<br>অন্যান্য______________ ....96<br>(নির্দিষ্ট কর ্ন)	
114	আপনার স্বামীর মাসে আনুমানিক কত টাকা আয় হয়?<br>যদি আয় টাকাতে না হয়ে চাল, গম বা অন্য কিছুতে হয় তবে তার পরিমাণ উলে- খ কর ্ন।<br>**না হলে 000 লিখুন।**	আয়................. [ ][ ][ ][ ][ ] টাকা<br>চাল .... [ ][ ][ ]. কেজি.<br>গম.................... [ ][ ][ ] কেজি<br>অন্যান্য______________ ....996<br>(নির্দিষ্ট কর ্ন)	

Section B: Reproduction and Birth History

আপনার জীবনে আপনি যতবার গর্ভধারণ করেছেন, সেই সব গর্ভ সম্পর্কে এখন আমি আপনাকে কিছু প্রশ্ন জিজ্ঞাসা করতে চাই।

No.	QUESTIONS AND FILTERS	CODING CATEGORIES	SKIP
201	আপনার কি কখনও কোন ছেলেমেয়ে হয়েছে?	হ্যাঁ ....1<br>না 2	 → 206
202	আপনার মোট কয়জন ছেলে এবং কয়জন মেয়ে হয়েছে?<br>**ছেলে মেয়ে না হলে 00 লিখুন।**	ছেলে .... ☐☐<br>মেয়ে .... ☐☐	
203	আপনার মোট কয়জন ছেলে এবং কয়জন মেয়ে জীবিত?<br>**ছেলে মেয়ে জীবিত না থাকলে 00 লিখুন।**	ছেলে .... ☐☐<br>মেয়ে .... ☐☐	
204	আপনি কি কখনো এমন কোন ছেলে বা মেয়ে জন্ম দিয়েছেন, যে জীবিত জন্ম নিয়েছিল কিন্তু জন্ম নেওয়ার পর মারা গিয়েছিল?<br>**যদি না হয়, যাচাই করুনঃ এমন কোন ছেলে বা মেয়ে, যে জন্ম নেয়ার পর কেঁদেছিল বা যার মধ্যে জীবনের লক্ষণ দেখা গিয়েছিল, কিন্তু কয়েক ঘন্টা বা কয়েক দিন মাত্র জীবিত ছিল অর্থাৎ পরে মারা গিয়েছিল?**	হ্যাঁ ....1<br>না 2	 → 206
205	সর্বমোট কয়জন ছেলে এবং কয়জন মেয়ে মারা গিয়েছে?<br>**ছেলে মেয়ে মারা না গেলে 00 লিখুন।**	ছেলে, মারা গেছে .... ☐☐<br>মেয়ে, মারা গেছে .... ☐☐	
206	কোন কোন গর্ভাবস্থা পূর্ণ মেয়াদের আগেই গর্ভনষ্ট (মিসক্যারেজ) গর্ভপাত (এ্যাবরশন), বা এম আর হিসাবে শেষ হয়ে যেতে পারে। আবার কোন কোন গর্ভাবস্থা মৃতজন্ম (Still birth) বা মৃত শিশুর জন্মও দিতে পারে অর্থাৎ যার জন্মের সময় জীবনের কোন লক্ষনই থাকে না। আপনার জীবনে কি কখনও এ ধরনের কোন ঘটনা অর্থাৎ জীবিত বাচ্চা জন্ম না দেয়ার মত ঘটনা ঘটেছে?	হ্যাঁ ....1<br>না 2	 → 208
207	মোট কতগুলো গর্ভাবস্থার ক্ষেত্রে জীবিত বাচ্চা জন্ম না দেয়ার মত ঘটনা ঘটেছে?<br>**প্রতিটি প্রশ্ন আলাদা করে জিজ্ঞেস করুন।**<br><br>এর মধ্যে কতটি গর্ভনষ্ট (মিসক্যারেজ)?<br>এর মধ্যে কতটি গর্ভপাত (এ্যাবরশন)?<br>এর মধ্যে কতটি এম, আর?<br>এর মধ্যে কতটি মৃতজন্ম?<br>**উত্তর না হলে, '00' লিখুন।**<br>**সবগুলো উত্তর যোগ করে মোটের সাথে মিলিয়ে দেখুন। অসামঞ্জস্য হলে পুনরায় প্রশ্ন জিজ্ঞেস করে সংশোধন করুন।**	মোট জীবিত বাচ্চা জন্ম না দেয়ার<br>সংখ্যা .... ☐☐<br><br>গর্ভনষ্ট (মিসক্যারেজ) .... ☐☐<br>গর্ভপাত (এ্যাবরশন)..................... ☐☐<br>এম, আর .... ☐☐<br>মৃতজন্ম .... ☐☐	
208	**সাক্ষাৎকারগ্রহণকারীঃ প্রশ্ন 203, 205 এবং 207 এর মোট বাচ্চা জন্ম দেয়ার সংখ্যা যোগ করুন এবং পাশের বক্সে লিখুন।**	মোট গর্ভের সংখ্যা .... ☐☐	

No.	QUESTIONS AND FILTERS	CODING CATEGORIES		SKIP
209	**সাক্ষাৎকারগ্রহণকারীঃ প্রশ্ন 208 দেখুন এবং সঠিক কোড বৃত্তায়িত করুন।**	মোট গর্ভের সংখ্যা 01 বা তার অধিক .... 1<br>মোট গর্ভের সংখ্যা 00 2		2 → **1026**
210	**সাক্ষাৎকারগ্রহনকারীঃ 208 দেখুন এবং মোট গর্ভের সংখ্যা নির্দিষ্ট স্থানে লিখে উত্তরদাতাকে প্রশ্নটি জিজ্ঞেস করুন। আপনি সারাজীবনে মোট কতবার গর্ভধারণ করেছেন, সেই সংখ্যা সঠিকভাবে লিখেছি কি-না এ বিষয়ে নিশ্চিত হওয়ার জন্য আমি আপনাকে আবারও জিজ্ঞেস করতে চাই, আপনি মোট________ বার গর্ভধারণ করেছিলেন, এটা কি ঠিক?**<br>(গর্ভের সংখ্যা)<br>হ্যাঁ ☐ ↓ না ☐ →	**201 থেকে 207 প্রশ্নের উত্তরগুলো দেখুন এবং প্রয়োজনে সংশোধন করুন।**		

সাক্ষাৎকারগ্রহণকারীঃ ০১ নভেম্বর ২০১১ থেকে ৩১ জানুয়ারী ২০১৩ এর মধ্যে উত্তরদাতার সর্বশেষ গর্ভ সম্পর্কে জিজ্ঞেস করতে হবে, সুতরাং উত্তরদাতাকে সেই গর্ভ সম্পর্কে ভাল করে বুঝিয়ে তারপর প্রশ্ন জিজ্ঞেস করুন।

এবার আমি আপনার ____গর্ভের ব্যাপারে আলোচনা করতে চাই।
(০১ নভেম্বর ২০১১ থেকে ৩১ জানুয়ারী ২০১৩ এর মধ্যে হওয়া সর্বশেষ গর্ভ)
সেই গর্ভের বা গর্ভাবস্থা থেকে জীবিত বাচ্চা বা মৃত বাচ্চা যাই জন্ম নিক না কেন বা সেই গর্ভ যদি মেয়াদের আগে মিসক্যারেজ বা গর্ভপাত হয়ে থাকে তাহলে সে সম্পর্কেও আলোচনা করতে চাই।

আপনার, ০১ নভেম্বর ২০১১ থেকে ৩১ জানুয়ারী ২০১৩ এর মধ্যে হওয়া সর্বশেষ গর্ভাবস্থার কথা চিন্তা করুন।

No.	QUESTIONS AND FILTERS	CODING CATEGORIES		SKIP
211	কোন বছরের কোন মাসের কত তারিখে আপনার সেই গর্ভ শেষ হয়েছিল?	☐☐ দিন ☐☐ মাস ☐☐☐☐ সাল		
211a	এই গর্ভ কত মাস স্থায়ী হয়েছিল?<br>**(পূর্ণ মাসের হিসাবে লিখুন)**	মাস .... ☐☐		
**212**	**সাক্ষাৎকারগ্রহণকারীঃ প্রশ্ন 211a দেখুন এবং সঠিক কোড বৃত্তায়িত করুন ।**	**03 মাস বা তার কম.......................................1**<br>**03 মাসের বেশি...2**		**1 → 800**
213	এই গর্ভে একটি না-কি একাধিক বাচ্চা ছিল?	একটি ....1<br>একাধিক ....2<br>জানি না/মনে নাই 7		

যমজ বাচ্চার ক্ষেত্রে আলাদা কলাম ব্যবহার করুন । তার চেয়ে বেশী সংখ্যক বাচ্চা হলে আলাদা প্রশ্নপত্রে সেই বাচ্চার তথ্য সংগ্রহ করুন এবং এই প্রশ্নপত্রের সাথে যুক্ত করুন । নির্দিষ্ট কলামে বাচ্চার নাম লিখুন এবং নাম উলে- খ করে প্রশ্ন করুন।

No.	QUESTIONS AND FILTERS	**বাচ্চা 1**	**বাচ্চা 2**	SKIP
214	আপনার এই গর্ভের ফলাফল কি ছিল?<br>জীবিত জন্ম না মৃত নাকি মেয়াদের আগে নষ্ট হওয়া গর্ভ (যেমনঃ গর্ভনষ্ট, গর্ভপাত, মিসক্যারেজ বা এম আর)?	জীবিত জন্ম .... 1<br>**(216 এ যান)**<br>মৃত জন্ম .... 2<br>৭ মাসের আগে নষ্ট .....3<br>**(800 এ যান)**	জীবিত জন্ম .... 1<br>**(216 এ যান)**<br>মৃত জন্ম .... 2<br>৭ মাসের আগে নষ্ট .....3<br>**(800 এ যান)**	
215	জন্মের পর বাচ্চাটি কেঁদেছিল বা নড়াচড়া করেছিল বা শ্বাস-প্রশ্বাস (দম/উয়া) নিয়েছিল কি?	হ্যাঁ.......................1<br>না........................2<br>**(220 এ যান)**	হ্যাঁ.......................1<br>না........................2<br>**(220 এ যান)**	
216	বাচ্চাটির নাম কি রাখা হয়েছিল?<br>**যদি নাম না রাখা হয়ে থাকে তবে "নাম রাখা হয়নি" লিখুন।**	নামঃ_______________	নামঃ_______________	
216a	_____ছেলে না-কি মেয়ে?<br>(নাম)	ছেলে ....1<br>মেয়ে 2	ছেলে .... 1<br>মেয়ে 2	
217	_____কি বর্তমানে জীবিত ?<br>(নাম)	জীবিত ....1<br>মৃত ....2<br>**(219 এ যান)**	জীবিত .... 1<br>মৃত .... 2<br>**(219 এ যান)**	

No.	QUESTIONS AND FILTERS	CODING CATEGORIES		SKIP
218	_____ এর বর্তমানে বয়স কত?<br>(নাম)<br>**(পূর্ণ মাসের হিসাবে বয়স লিখুন)**	বাচ্চা 1<br>মাস ....[ ][ ]<br>(220 এ যান)	বাচ্চা 2<br>মাস ....[ ][ ]<br>(220 এ যান)	
		**(প্রশ্ন 211 এর সাথে 218 এর বয়স মিলিয়ে দেখুন, অসামঞ্জস্য হলে প্রয়োজনে সংশোধন করন)**		
219	জীবিত জন্ম কিন্তু বর্তমানে মৃত হলে জিজ্ঞেস কর‍ুনঃ মৃত্যুর সময় তার বয়স কত ছিল?<br>**মৃত্যুর সময় বাচ্চার বয়স ১ মাসের কম হলে উত্তর দিনে রেকর্ড কর‍ুন। এবং জিজ্ঞেস কর‍ুনঃ** মৃত্যুর সময় ___ এর<br>(নাম)<br>বয়স কত দিন ছিল? **(দিন সম্পর্কে ভালভাবে প্রোব কর‍ুন)**<br>**মৃত্যুর সময় বাচ্চার বয়স ১ মাসের বেশি হলে উত্তর মাসে রেকর্ড কর‍ুন এবং জিজ্ঞেস কর‍ুনঃ** মৃত্যুর সময় ___এর<br>(নাম)<br>বয়স কয় মাস ছিল**? (মাস সম্পর্কে ভালভাবে প্রোব কর‍ুন)**।<br>**মৃত্যুর সময় বয়স ১ দিনের কম হলে দিনের ঘরে 00 লিখুন।**	দিন [ ][ ]<br>মাস [ ][ ]	দিন [ ][ ]<br>মাস [ ][ ]	
220	___যখন আপনার গর্ভে এসেছিল, তখনই কি গর্ভবতী<br>(নাম)<br>হতে চেয়েছিলেন, না কি অপেক্ষা করে পরে গর্ভবতী হতে চেয়েছিলেন না কি একেবারেই কোন (আর কোন) ছেলে মেয়ে চান নি?	তখনই চেয়েছিলাম .... 1<br>অপেক্ষা করে পরে গর্ভবতী হতে চেয়েছিলাম .... 2<br>একেবারেই আর কোন ছেলে মেয়ে চাই নি.................. 3		
221	আপনি কি কখনও আপনার রক্তচাপ/ব-াড প্রেসার একজন ডাক্তার বা স্বাস্থ্যকর্মী দ্বারা মেপেছিলেন?	হ্যাঁ.. 1<br>না.. 2<br>মনে নাই.. 7		2, 7 → **225**
222	কোন ডাক্তার বা স্বাস্থ্যকর্মী আপনাকে কখনও বলেছেন কি যে আপনার উচ্চ রক্তচাপ/**হাই** ব-াড প্রেসার আছে?	হ্যাঁ.. 1<br>না.. 2<br>মনে নাই.. 7		2, 7 → **225**
223	**প্রথম** কখন আপনার উচ্চ রক্তচাপ/**হাই** ব-াড প্রেসার ধরা পড়েছে?	গর্ভাবস্থায়.. 1<br>যখন গর্ভে কোন সন্তান ছিল না............................ 2<br>মনে নাই.. 7		
224	উচ্চ রক্তচাপের/**হাই** ব-াড প্রেসার এর জন্য কখনো কোন ঔষধ খেয়েছেন কি?	হ্যাঁ.. 1<br>না.. 2<br>মনে নাই.. 7		
225	আপনি কি কখনো আপনার ডায়াবেটিস/রক্তের সুগার কোন ডাক্তার বা স্বাস্থ্যকর্মী দ্বারা মাপিয়েছিলেন?	হ্যাঁ.. 1<br>না.. 2<br>মনে নাই.. 7		2, 7 → **301**
226	কোন ডাক্তার বা স্বাস্থ্যকর্মী কি কখনও আপনাকে বলেছেন, আপনার ডায়াবেটিস/রক্তে সুগার বেশী আছে?	হ্যাঁ.. 1<br>না.. 2<br>মনে নাই.. 7		2, 7 → **301**
227	**প্রথম** কখন আপনার ডায়াবেটিস/রক্তে সুগার বেশী ধরা পড়েছে?	গর্ভাবস্থায়.. 1<br>যখন গর্ভে কোন সন্তান ছিল না............................ 2<br>মনে নাই.. 7		
228	ডায়াবেটিস/রক্তে সুগার বেশীর জন্য কখনো ইনসুলিন নিয়েছেন বা ডায়াবেটিস এর কোন ঔষধ খেয়েছেন কি?	হ্যাঁ.. 1<br>না.. 2<br>মনে নাই.. 7		

Section C: Antenatal Care

সাক্ষাৎকারগ্রহণকারীঃ ০১ নভেম্বর ২০১১ থেকে ৩১ জানুয়ারী ২০১৩ এর মধ্যে উত্তরদাতার সর্বশেষ গর্ভের গর্ভকালীন যত্ন সম্পর্কে জিজ্ঞেস করতে হবে, সুতরাং উত্তরদাতাকে সেই গর্ভ সম্পর্কে ভাল করে বুঝিয়ে তারপর প্রশ্ন জিজ্ঞেস করুন।

NO.	QUESTIONS AND FILTERS	CODING CATEGORIES	SKIP
301	_____পেটে থাকাকালীন সময়ে/এই গর্ভকালীন সময়ে<br>(নাম)<br>চেকআপ করার জন্য আপনি কোন স্বাস্থ্যকর্মীর কাছে গিয়েছিলেন কি?<br>**সাক্ষাৎকারগ্রহনকারীঃ উত্তরদাতাকে বুঝিয়ে বলুন স্বাস্থ্যকর্মী বলতে ডাক্তারসহ সব ধরনের স্বাস্থ্যকর্মীর কথাই আপনি জানতে চাচ্ছেন।**	হ্যাঁ .... 1<br>না 2	2→ **306**
302	এই গর্ভকালীন চেকআপের জন্য স্বাস্থ্যকর্মীর কাছে আপনি মোট কতবার গিয়েছিলেন?	বার .... [ ][ ]<br>জানিনা/মনে নাই 97	
303	এই গর্ভকালীন চেকআপ করার জন্য আপনি কোন্ কোন্ স্বাস্থ্যকর্মীর কাছে গিয়েছিলেন?<br><br>**উত্তর পড়ে শোনাবেন না।**<br><br>**একাধিক উত্তর হতে পারে।**	পাশ করা (MBBS) ডাক্তার.............................. A<br>নার্স/ধাত্রী .... B<br>প্যারামেডিক... C<br>পরিবার কল্যাণ পরিদর্শিকা (FWV) .... D<br>কম্যুউনিটি স্কিল্ড বার্থ এটেন্টডেন্ট (CSBA) ....E<br>উপসহকারী কমিউনিটি চিকিৎসা কর্মকর্তা (সাকমো) ....F<br>মা-মনি স্বাস্থ্যকর্মী/CHW .... G<br>স্বাস্থ্য সহকারী (HA) .... H<br>পরিবার কল্যাণ সহকারী (FWA) ....I<br>কমিউনিটি হেলথ কেয়ার প্রোভাইডার (CHCP).......... J<br>প্রশিক্ষণপ্রাপ্ত টিবিএ TTBA .... K<br>প্রশিক্ষণহীন টিবিএ (ধন্নী, চাউনি, দাই) ....L<br>হোমিওপ্যাথ... M<br>আয়ুর্বেদিক চিকিৎসক .... N<br>হাতুরে ডাক্তার/কোয়াক .... O<br>গ্রাম ডাক্তার/পল্লী চিকিৎসক................................P<br>ওঝা/ কবিরাজ .... Q<br>অন্যান্য স্বাস্থ্যকর্মী.. R<br>অন্যান্য______________ .... X<br>(নির্দিষ্ট করুন)<br>জানি না/মনে নাই .... Y	

304	এই গর্ভকালীন **চেকআপের** সময়ে কখনও কি আপনার _____ হয়েছিল? **প্রত্যেকটি চেকআপের বিষয় পড়ে শোনান।**<br>(বিষয়)	বিষয়	হ্যাঁ	না	
	ওজন নেয়া?	ওজন নেয়া.....................................	1	2	
	ব্লাড প্রেসার মাপা?	ব্লাড প্রেসার মাপা	1	2	
	প্রস্রাব পরীক্ষা করা?	প্রস্রাব পরীক্ষা করা	1	2	
	রক্ত পরীক্ষা করা?	রক্ত পরীক্ষা করা	1	2	
	আলট্রাসনোগ্রাম করা?	আলট্রাসনোগ্রাম করা..........................	1	2	
	আপনার পেটে হাত দিয়ে পরীক্ষা করা?	আপনার পেটে হাত দিয়ে পরীক্ষা কর	1	2	

NO.	QUESTIONS AND FILTERS	CODING CATEGORIES	SKIP
305	এই গর্ভের সময় প্রথম যখন আপনি গর্ভকালীন চেকআপ করিয়েছিলেন, তখন আপনি কত মাসের গর্ভবতী ছিলেন?	মাস .... [ ][ ]<br>জানি না 97	→ 307
306	আপনি কেন গর্ভকালীন চেকআপ এর জন্য কাউকে দেখান নি?<br><br>**জিজ্ঞেস করুন আরও কিছু?**<br><br>**উত্তরের কোড বৃত্তায়িত করুন।**<br><br>**একাধিক উত্তর হতে পারে।**	চেকআপ এর প্রয়োজন ছিল বলে মনে হয় নি............. A<br>জানতাম না কোথায় যেতে হবে .... B<br>অনেক খরচ/ টাকা পয়সা ছিল না .... C<br>স্বাস্থ্যকেন্দ্র বাসা হতে অনেক দুরে.......................... D<br>যানবাহনের সমস্যা... E<br>সাথে যাবার মত কেউ ছিল না .... F<br>স্বাস্থ্যকেন্দ্রে যাবার মত সময় ছিল না .... G<br>স্বাস্থ্যকেন্দ্র বন্ধ ছিল/কোন স্বাস্থ্যকর্মী ছিলেন না............H<br>স্বাস্থ্যকেন্দ্রের সেবা অনুন্নত মানের .... I<br>স্বাস্থ্যকেন্দ্রের সেবাদানকারীদের ব্যবহার খারাপ .... J<br>চেকআপ এর জন্য স্বাস্থ্যকেন্দ্রে অনেকক্ষন<br>বসে থাকতে হয়... K<br>স্বাস্থ্যকেন্দ্রে ঔষধ পত্র পাওয়া যায় না....................... L<br>অন্যান্য ________________ .... X<br>(নির্দিষ্ট করুন)	
307	_____পেটে থাকাকালীন সময়ে/এই গর্ভকালীন<br>(নাম)<br>সময়ে আপনার কি ম্যালেরিয়া হয়েছিল?	হ্যাঁ ....1<br>না ....2<br>মনে নাই...7	2, 7 → 309
308	এই ম্যালেরিয়ার জন্য আপনি কি কোন চিকিৎসা করিয়েছিলেন?	হ্যাঁ .... 1<br>না .... 2<br>মনে নাই.. 7	
309	_____ পেটে থাকাকালীন সময়ে/এই গর্ভকালীন<br>(নাম)<br>সময়ে কোন ডাক্তার/স্বাস্থ্যকর্মী বা কেউ কি কখনও আপনাকে বলেছেন, আপনার ভিটামিন এর স্বল্পতা আছে?	হ্যাঁ ....1<br>না ....2<br>মনে নাই...7	2, 7 → 311
310	ডাক্তার/স্বাস্থ্যকর্মী কোন ভিটামিন এর স্বল্পতার কথা বলেছিল?	ভিটামিন এ...A<br>ভিটামিন বি...B<br>ভিটামিন সি ....D<br>ভিটামিন ডি ....E<br>ভিটামিন ই .... F<br>ভিটামিন কে...G<br>মনে নাই...Y	
311	_____ পেটে থাকাকালীন সময়ে/এই গর্ভকালীন সময়ে<br>(নাম)<br>আপনার কখনো রাতকানা রোগ হয়েছিল কি বা কোন ডাক্তার/স্বাস্থ্যকর্মী বা কেউ কি কখনও আপনাকে বলেছেন, আপনার রাতকানা রোগ আছে?	হ্যাঁ .... 1<br>না ....2<br>মনে নাই 7	
312	_____ পেটে থাকাকালীন সময়ে/এই গর্ভকালীন<br>(নাম)<br>সময়ে কোন ডাক্তার বা স্বাস্থ্যকর্মী কি কখনও আপনাকে বলেছেন, আপনার রক্তস্বল্পতা আছে?	হ্যাঁ .... 1<br>না ....2<br>মনে নাই 7	
313	আপনার এই গর্ভকালীন সময়ে আপনি মোট কয়টি টিটেনাস ইনজেকশন নিয়েছিলেন, যা মা ও শিশুকে খিচুনী থেকে রক্ষা করে?	সংখ্যা... .... [ ]<br>কোন টিটেনাস ইনজেকশন নেই নাই........... ....6<br>মনে নাই/ জানি না 7	

NO.	QUESTIONS AND FILTERS	CODING CATEGORIES	SKIP
314	এই গর্ভের আগে আপনি মোট কয়টি টিটেনাস ইনজেকশন নিয়েছিলেন?	সংখ্যা... [ ]<br>কোন টিটেনাস ইনজেকশন নেই নাই.......................6<br>মনে নাই/ জানি না..7	
315	এই গর্ভকালীন সময়ে আপনি আয়রন ট্যাবেলেট (মাইট্যা ট্যাবলেট) বা আয়রন সিরাপ খেয়েছিলেন কি যা শরীরে রক্ত হওয়ার জন্য মায়েরা খায়?	হ্যাঁ ....1<br>না ....2<br>জানি না..7	2, 7 → 319
316	আপনি গর্ভের কত মাস থেকে আয়রন ট্যাবলেট/সিরাপ খাওয়া শুর‍ু করেছিলেন?	মাস .... [ ][ ]<br>জানি না/মনে নাই 97	
317	এই গর্ভকালীন সময়ে আপনি মোট কত মাস আয়রন ট্যাবলেট/সিরাপ খেয়েছিলেন?<br>**১ মাসের কম হলে দিনে লিখুন।**	মাস .... 1 [ ][ ]<br>দিন.. 2 [ ][ ]<br>মনে নাই/ জানি না 997	
318	আপনি এই গর্ভকালীন সময়ে মোট কয়টি আয়রন ট্যাবলেট/ সিরাপের ফাইল/বোতল খেয়েছেন?	সংখ্যা (ট্যাবলেট).................... [ ][ ][ ]<br>সংখ্যা (ফাইল/বোতল)...................... [ ][ ]<br>মনে নাই/ জানি না.......................................997	
319	_____পেটে থাকাকালীন সময়ে/এই গর্ভকালীন<br>(নাম)<br>সময়ে আপনার ওজন গর্ভের শেষ তিন মাসে কতটুকু বেড়েছিল?	স্বাভাবিক বৃদ্ধির চেয়ে বেশি ....1<br>স্বাভাবিক বৃদ্ধি..2<br>স্বাভাবিক বৃদ্ধির চেয়ে কম....................................3<br>জানি না/মনে নাই...7	
320	এই গর্ভকালীন সময়ে আপনার কোন সমস্যা/অসুবিধা/জটিলতা হয়েছিল কি যার জন্য চিকিৎসার প্রয়োজন ছিল?	হ্যাঁ ....1<br>না ....2<br>মনে নাই...7	2, 7 → **401**

NO.	QUESTIONS AND FILTERS	CODING CATEGORIES		SKIP
	এখন আমি আপনাকে আপনার গর্ভকালীন সময়ের সমস্যা/অসুবিধা/জটিলতার কথা জিজ্ঞেস করব।			
321.	আপনার কি ধরণের সমস্যা/অসুবিধা/জটিলতা হয়েছিল?<br>**জিজ্ঞেস করুন: আরও কিছু?**	321a.	**321 প্রশ্নে যে সমস্যার কোড বৃত্তায়িত হবে, 321a প্রশ্নে সেই সমস্যা সম্পর্কেই জিজ্ঞেস করুন।**<br>**_________ এর জন্য মোট কত দিন অসুস্থ ছিলেন?**<br>(সমস্যা/অসুবিধা)<br>**১ দিনের কম হলে 00 লিখুন।** দিন	
	তীব্র মাথা ব্যথা.................................... A		তীব্র মাথা ব্যথা.......................... [ ][ ]	
	চোখে ঝাপসা দেখা.................................. B		চোখে ঝাপসা দেখা...................... [ ][ ]	
	বাচ্চা হওয়ার রাস্ড়ে অতিরিক্ত রক্তস্রাব C		বাচ্চা হওয়ার রাস্ড়ে অতিরিক্ত রক্তস্রাব . [ ][ ]	
	জ্বর .... D		জ্বর .... [ ][ ]	
	খিঁচুনী/ফিট .... E		খিঁচুনী/ফিট............................... [ ][ ]	
	হাতে পানি আসা/ফুলে যাওয়া...................... F		হাতে পানি আসা/ফুলে যাওয়া.......... [ ][ ]	
	মুখমন্ডলে পানি আসা/ফুলে যাওয়া .... G		মুখমন্ডলে পানি আসা/ফুলে যাওয়া...... [ ][ ]	
	গর্ভের বাচ্চার নড়াচড়া কমে যাওয়া/বন্ধ হওয়া.... H		গর্ভের বাচ্চার নড়াচড়া কমে যাওয়া/বন্ধ [ ][ ]	
	তলপেটে তীব্র ব্যথা I		তলপেটে তীব্র ব্যথা...................... [ ][ ]	
	পায়ে পানি আসা...................................... J		পায়ে পানি আসা.......................... [ ][ ]	
	উচ্চ রক্তচাপ.. K		উচ্চ রক্তচাপ.............................. [ ][ ]	
	ডায়াবেটিস L		ডায়াবেটিস................................ [ ][ ]	
	সময় পূর্ণ হওয়ার আগে পানি ভাঙ্গা .... M		সময় পূর্ণ হওয়ার আগে পানি ভাঙ্গা..... [ ][ ]	
	অচেতন হওয়া/জ্ঞান হারিয়ে ফেলা................. N		অচেতন হওয়া/জ্ঞান হারিয়ে ফেলা...... [ ][ ]	
	কষ্ট করে শ্বাস নেয়া.................................. O		কষ্ট করে শ্বাস নেয়া...................... [ ][ ]	
	প্রচন্ড দুর্বলতা... P		প্রচন্ড দুর্বলতা............................. [ ][ ]	
	অতিরিক্ত বমি .... Q		অতিরিক্ত বমি .... [ ][ ]	
	অতিরিক্ত সাদা স্রাব.................................. R		অতিরিক্ত সাদা স্রাব...................... [ ][ ]	
	**অন্যান্য**__________................................ X<br>(নির্দিষ্ট করুন)		**অন্যান্য**__________.................. [ ][ ]<br>**(নির্দিষ্ট করুন)**	
322	_________ **এর** জন্য আপনি কি কোন চিকিৎসা<br>(321 এর উত্তর)<br>করিয়েছেন?	হ্যাঁ .... 1<br>না .... 2<br>জানি না/মনে নেই... 7		→ **324**

NO.	QUESTIONS AND FILTERS	CODING CATEGORIES	SKIP
323	আপনি কার কাছে চিকিৎসা করিয়েছেন?<br><br>**জিজ্ঞেস করুনঃ আরও কার কাছে গিয়েছিলেন?**<br><br>**সব উত্তরের কোড বৃত্তায়িত করুন।**<br><br>**একাধিক উত্তর হতে পারে।**	পাশ করা (MBBS) ডাক্তার.......... A<br>নার্স/ধাত্রী.......... B<br>প্যারামেডিক.......... C<br>পরিবার কল্যাণ পরিদর্শিকা (FWV).......... D<br>কম্যুউনিটি স্কিল্ড বার্থ এটেন্টডেন্ট (CSBA).......... E<br>উপসহকারী কমিউনিটি চিকিৎসা কর্মকর্তা (সাকমো)..... F<br>মা-মনি স্বাস্থ্যকর্মী/CHW.......... G<br>স্বাস্থ্য সহকারী (HA).......... H<br>পরিবার কল্যাণ সহকারী (FWA).......... I<br>কমিউনিটি হেলথ কেয়ার প্রোভাইডার (CHCP).......... J<br>প্রশিক্ষণপ্রাপ্ত টিবিএ (TTBA).......... K<br>প্রশিক্ষণহীন টিবিএ (ধন্নী, চাউনি, দাই).......... L<br>হোমিওপ্যাথ.......... M<br>আয়ুর্বেদিক চিকিৎসক.......... N<br>হাতুরে ডাক্তার/কোয়াক.......... O<br>গ্রাম ডাক্তার/পল-ী চিকিৎসক.......... P<br>ওঝা/ কবিরাজ.......... Q<br>অন্যান্য স্বাস্থ্যকর্মী.......... R<br>অন্যান্য___________ .... X<br>(নির্দিষ্ট করুন)<br>জানি না/মনে নাই.......... Y	→ **401**
324	________এর জন্য কেন আপনি চিকিৎসা করান নি?<br>(321 এর উত্তর)<br><br>**জিজ্ঞেস করুন: আরও কিছু?**<br><br>**সব উত্তরের কোড বৃত্তায়িত করুন।**<br><br>**একাধিক উত্তর হতে পারে।**	চিকিৎসার প্রয়োজন আছে বলে মনে হয় নি.......... A<br>জানতাম না কোথায় যেতে হবে.......... B<br>অনেক খরচ/ টাকা পয়সা ছিল না.......... C<br>স্বাস্থ্যকেন্দ্র বাসা হতে অনেক দুরে.......... D<br>যানবাহনের সমস্যা.......... E<br>সাথে যাবার মত কেউ ছিল না.......... F<br>স্বাস্থ্যকেন্দ্রে যাবার মত সময় ছিল না.......... G<br>স্বাস্থ্যকেন্দ্র বন্ধ ছিল/কোন স্বাস্থ্যকর্মী ছিলেন না.......... H<br>স্বাস্থ্যকেন্দ্রের সেবা অনুন্নত মানের.......... I<br>স্বাস্থ্যকেন্দ্রের সেবাদানকারীদের ব্যবহার খারাপ.......... J<br>চিকিৎসা পাবার জন্য স্বাস্থ্যকেন্দ্রে অনেকক্ষন<br>বসে থাকতে হয়.......... K<br>স্বাস্থ্যকেন্দ্রে ঔষধ পত্র পাওয়া যায় না.......... L<br>অন্যান্য ________________ .... X<br>**(নির্দিষ্ট করুন)**	

Section D: Delivery

এখন আমি আপনার ০১ নভেম্বর ২০১১ থেকে ৩১ জানুয়ারী ২০১৩ এর মধ্যে হওয়া সর্বশেষ ডেলিভারি সম্পর্কে কিছু প্রশ্ন জিজ্ঞেস করব।

NO.	QUESTIONS AND FILTERS	CODING CATEGORIES	SKIP
401	প্রসব ব্যথা শুরু হওয়ার কতক্ষন পর আপনার ডেলিভারি হয়েছিল?	মিনিট .... 1 [ ][ ]<br>ঘন্টা .... 2 [ ][ ]<br>দিন................................... 3 [ ][ ]<br>প্রসব ব্যথা হয় নি .... 995<br>মনে নেই 997	995 → **403**
402	প্রসব ব্যথা শুরু হওয়ার পূর্বে না -কি পরে পানি ভেঁঙ্গেছিল?	প্রসব ব্যথার পূর্বে .... 1<br>প্রসব ব্যথার পরে .... 2<br>একই সাথে .... 3<br>পানি ভাঙ্গে নি.. 4<br>জানি না 7	3, 4, 7 → **404**
403	প্রসবের কতক্ষন পূর্বে/পরে পানি ভেঁঙ্গেছিল?	মিনিট .... 1 [ ][ ]<br>ঘন্টা................................. 2 [ ][ ]<br>দিন................................... 3 [ ][ ]<br>পানি ভাঙ্গে নি..995<br>মনে নেই 997	
404	আপনার কি ধরণের ডেলিভারি হয়েছিল?	স্বাভাবিক/নরমাল ডেলিভারি................................. 1<br>সিজারিয়ান/ পেট কেটে অপারেশন.......................... 2<br>যন্ত্রের সাহায্যে ডেলিভারি .... 3<br>অন্যান্য ____________________ ....6<br>(নির্দিষ্ট করুন)	1 → **406**
405	আপনার ডেলিভারি __________ এর মাধ্যমে কেন<br>(404 এর উত্তর)<br>হয়েছিল?	আগের বাচ্চা সিজারিয়ান এর মাধ্যমে হয়েছিল .... 01<br>গর্ভে শিশুর অস্বাভাবিক অবস্থান ছিল....................... 02<br>শিশুর হাত পা আগে বের হয়ে এসেছিল .... 03<br>খিঁচুনী হয়েছিল... 04<br>দীর্ঘ প্রসব (১২ ঘন্টার বেশি) ব্যথা ছিল .... 05<br>আমি চেয়েছিলাম .... 06<br>কোন সমস্যা ছিল না তবুও ডাক্তার করতে বলেছিল ..... 07<br>প্রসব ব্যথা ছিল না .... 08<br>অন্যান্য ____________________ .... 96<br>(নির্দিষ্ট করুন)<br>জানি না/বলতে পারি না 97	

NO.	QUESTIONS AND FILTERS				CODING CATEGORIES	SKIP
	এখন আমি আপনাকে আপনার ডেলিভারির সময়ের কিছু শারীরিক সমস্যার কথা জিজ্ঞেস করব।					
406	ডেলিভারির সময় কি আপনার___________ (সমস্যা/জটিলতা) হয়েছিল? (প্রত্যেকটি সমস্যা/জটিলতা সম্পর্কে জিজ্ঞেস কর¯^ন)	হ্যাঁ	না	406a.	**406 প্রশ্নে যে যে সমস্যার কোড 1 বৃত্তায়িত হবে, 406a প্রশ্নে সেই সমস্যা সম্পর্কেই জিজ্ঞেস কর¯^ন।** _________ এর জন্য মোট কত দিন অসুস্থ ছিলেন? (সমস্যা/অসুবিধা) **(১ দিনের কম হলে 00 লিখুন)** দিন	
a	বাচ্চা হওয়ার রাস্ড়া (যোনী পথে) দিয়ে অতিরিক্ত রক্ত গিয়েছিল	1	2		বাচ্চা হওয়ার রাস্ড়া (যোনী পথে) দিয়ে অতিরিক্ত রক্ত গিয়েছিল....................	
b	দুর্গন্ধযুক্ত স্রাব গিয়েছিল	1	2		দুর্গন্ধযুক্ত স্রাব গিয়েছিল....................	
c	তীব্র জ্বর হয়েছিল	1	2		তীব্র জ্বর হয়েছিল..........................	
d	শিশুর হাত পা আগে বের হয়ে এসেছিল	1	2			
e	(পেটের মধ্যে) শিশুর অস্বাভাবিক অবস্থান ছিল ..	1	2			
f	দীর্ঘ প্রসব (১২ ঘন্টার বেশি) ব্যথা ছিল....	1	2		দীর্ঘ প্রসব (১২ ঘন্টার বেশি) ব্যথা ছিল ..	
g	প- াসেন্টা বা ফুল পড়ে নি..................	1	2		প- াসেন্টা বা ফুল পড়ে নি................	
h	বাচ্চা থাকার থলি বা ইউটেরাস বা গর্ভদানী ছিঁড়ে গিয়েছিল	1	2			
i	জন্ম দ্বার ছিঁড়ে গিয়েছিল	1	2			
j	(শিশুর) নাড়ী বেরিয়ে এসেছিল	1	2			
k	(শিশুর গলায়) নাড়ী পেঁচিয়ে গিয়েছিল	1	2			
l	(শিশু) মাথায় বা শরীরে কোথাও আঘাত পেয়েছিল......................................	1	2			
m	খিঁচুনী হয়েছিল	1	2		খিঁচুনী হয়েছিল..............................	
n	তীব্র মাথা ব্যথা হয়েছিল	1	2		তীব্র মাথা ব্যথা হয়েছিল....................	
o	বাচ্চা হওয়ার রাস্তা (যোনী পথে) দিয়ে সবুজাভ কিছু বের হয়েছিল..................	1	2		বাচ্চা হওয়ার রাস্তা (যোনী পথে) দিয়ে সবুজাভ কিছু বের হয়েছিল	
p	পা/মুখ ফুলে গিয়েছিল........................	1	2		পা/মুখ ফুলে গিয়েছিল	
q	আরও কোন সমস্যা হয়েছিল_________ (নির্দিষ্ট কর¯^ন)	1	2		আরও কোন সমস্যা হয়েছিল_________ (নির্দিষ্ট কর¯^ন)	
407	আপনার ডেলিভারি কোথায় করাবেন সেই ব্যাপারে আগে থেকে কোন পরিকল্পনা করেছিলেন কি?				হ্যাঁ .... 1<br>না.. 2	 → **408**
407a	আপনার ডেলিভারি কোথায় করাবেন বলে পরিকল্পনা করেছিলেন ?				বাড়িতে..01<br>মেডিকেল কলেজ হাসপাতাল.................................02<br>জেলা/সদর হাসপাতাল..03<br>সরকারী স্বাস্থ্যকেন্দ্র ....04<br>এনজিও হাসপাতাল/ স্বাস্থ্যকেন্দ্র ....05<br>প্রাইভেট হাসপাতাল/ ক্লিনিক ....06<br>অন্যান্য _____________ ....96<br>(নির্দিষ্ট কর¯^ন)	

NO.	QUESTIONS AND FILTERS	CODING CATEGORIES	SKIP
408	আপনার ডেলিভারি কোথায় হয়েছিল?	**বাড়ি**<br>নিজ বাড়ি, স্বামী/শ্বশুর বাড়ি................................11<br>বাবার বাড়ি..12<br>অন্য কোন বাড়ি ....13<br>**সরকারী স্বাস্থ্যকেন্দ্র**<br>মেডিকেল কলেজ হাসপাতাল ....21<br>জেলা/সদর হাসপাতাল ....22<br>মা ও শিশু স্বাস্থ্যকেন্দ্র ....23<br>উপজেলা স্বাস্থ্য কমপে- ক্স.................................24<br>ইউনিয়ন স্বাস্থ্য ও পরিবার কল্যাণ কেন্দ্র/<br>সাব সেন্টার/আরডি ....25<br>কমিউনিটি ক্লিনিক ....26<br>**এনজিও স্বাস্থ্যকেন্দ্র**<br>এনজিও হাসপাতাল..31<br>এনজিও স্থায়ী স্বাস্থ্যকেন্দ্র...................................32<br>প্রাইভেট হাসপাতাল/ ক্লিনিক................................41<br>অন্যান্য প্রাইভেট স্বাস্থ্যকেন্দ্র ....42<br>হাসপাতালে যাওয়ার পথে ডেলিভারী হয়েছে ....43<br>অন্যান্য ____________________ ....96<br>(নির্দিষ্ট করুন)	11–13 → **409**<br><br>43 → **410**<br>96 → **409**
408a	ডেলিভারির জন্য হাসপাতালে ভর্তি হয়ে আপনাকে কতদিন থাকতে হয়েছিল?	ঘন্টা...1 [ ][ ]<br>দিন..2 [ ][ ]	→ **501**
409	কেন আপনি হাসপাতালে ডেলিভারি করান নি?<br><br>**জিজ্ঞেস করুনঃ আরও কিছু?**<br><br>**সব উত্তরের কোড বৃত্তায়িত করুন।**<br><br>**উত্তর একাধিক হতে পারে।**	অনেক দূরে...A<br>সুবিধাজনক সময়ে সেবা দেওয়া হয় না.....................B<br>সেবা প্রদানকারীর ব্যবহার ভাল নয়.........................C<br>সেবা প্রদানকারী দক্ষ নয়....................................D<br>গোপনীয়তার অভাব...E<br>পর্যাপ্ত ওষুধপত্র পাওয়া যায় না...............................F<br>অনেকক্ষণ অপেক্ষা করতে হয়..............................G<br>ব্যয়বহুল...H<br>ধর্মীয় কারণ ....I<br>যাওয়া দরকার এটা বুঝতে পারি নি.......................... J<br>পরিবারের অনুমতি ছিল না..................................K<br>কোথায় ডেলিভারি হয় জানতাম না...........................L<br>প্রয়োজন হয় নি/**ছিল না** ....M<br>অন্যান্য _______________ ....X<br>(নির্দিষ্ট করুন)	

NO.	QUESTIONS AND FILTERS	CODING CATEGORIES	SKIP
410	আপনার ডেলিভারিতে কে কে সাহায্য করেছিল?<br><br>**জিজ্ঞেস করুনঃ আরও কিছু?**<br><br>**সব উত্তরের কোড বৃত্তায়িত করুন।**<br><br>**উত্তর একাধিক হতে পারে।**<br><br>**সাক্ষাৎকারগ্রহণকারীঃ ডেলিভারিতে প্রধানত যিনি সাহায্য করেছিলেন অর্থ্যাৎ যার ভূমিকা সবচেয়ে বেশি ছিল শুধুমাত্র তার নাম লিখুন।**<br><br>নামঃ_______________________________	মা/ শ্বাশুড়ী .... A<br>বোন/ননদ/জা.. B<br>চাচী/মামী/খালা/ফুফু/দাদী/নানী .... C<br>ভাগনি/ভাতিজী .... D<br>স্বামী ....E<br>অন্য কোন আত্মীয় ___________ ....F<br>প্রতিবেশী/বন্ধু .... G<br>**স্বাস্থ্য পেশাজীবিঃ**<br>পাশ করা (MBBS) ডাক্তার .... H<br>নার্স/ধাত্রী.. I<br>পরিবার কল্যাণ পরিদর্শিকা (FWV) ....J<br>কম্যুউনিটি স্কিল্ড বার্থ এটেন্টডেন্ট(CSBA) .... K<br>উপসহকারী কমিউনিটি চিকিৎসা কর্মকর্তা (সাকমো)........L<br>মা-মনি স্বাস্থ্যকর্মী/ কমিউনিটি স্বাস্থ্যকর্মী .... M<br>স্বাস্থ্য সহকারী (HA) .... N<br>পরিবার কল্যাণ সহকারী (FWA) .... O<br>অন্যান্য পেশাজীবিঃ<br>প্রশিক্ষণপ্রাপ্ত টিবিএ ....P<br>প্রশিক্ষণহীন টিবিএ (ধন্নী, চাউনি, দাই) .... Q<br>হোমিওপ্যাথ.. R<br>আয়ুর্বেদিক চিকিৎসক ....S<br>হাতুড়ে ডাক্তার/কোয়াক..T<br>গ্রাম ডাক্তার/পল্লী চিকিৎসক............................... U<br>অন্যান্য ______________ .... X<br>(নির্দিষ্ট করুন)<br>কেউ নয়.. Y	
411	ডেলিভারির সময় মা-মনি স্বাস্থ্যকর্মী/কমিউনিটি স্বাস্থ্যকর্মী (CHW) উপস্থিত ছিলেন কি?	হ্যাঁ .... 1<br>না .... 2<br>মনে নেই.. 7	
412	ডেলিভারিতে প্রধানত যিনি সাহায্য করেছিলেন, ডেলিভারির পূর্বে তিনি তার হাত সাবান দিয়ে ধুয়েছিলেন কি?	হ্যাঁ .... 1<br>না .... 2<br>জানি না 7	
413	আপনার কি ডেলিভারি ব্যাগ/কিট ছিল?	হ্যাঁ .... 1<br>না .... 2<br>জানি না/মনে নেই.. 7	2, 7 → **501**
414	ডেলিভারি ব্যাগ/কিট এর কি কি জিনিস আপনার ডেলিভারীর সময় ব্যবহার করা হয়েছিল?<br><br>**সাক্ষাতকারগ্রহণকারীঃ উত্তরদাতা স্বতঃস্ফূর্তভাবে যে সব জিনিসের নাম উল্লেখ করবেন তার কোড দ্বিতীয় কলামে বৃত্তায়িত করুন অতঃপর যেগুলি বলবেন না, ডেলিভারী কিটের জিনিসগুলি এক এক করে দেখান এবং জিজ্ঞেস করুন এটি ব্যবহার করা হয়েছিল কি-না, উত্তর হ্যাঁ হলে তৃতীয় কলামে, না হলে চতুর্থ কলামে কোড বৃত্তায়িত করুন।**	(see table below)	

ডেলিভারী কিটের জিনিস	স্বতঃস্ফূর্ত ভাবে বলেছে	দেখানোর পর হ্যাঁ বলেছে	জানি না/না
প্লাস্টিক সিট	1	2	3
ব্লেড	1	2	3
সুতা	1	2	3
সাবান	1	2	3

Section E: Postnatal Care

NO.	QUESTIONS AND FILTERS	CODING CATEGORIES	SKIP
501	ডেলিভারির সময়ে অতিরিক্ত রক্তক্ষরণ বন্ধ করার জন্য _____এর জন্মের আগে আপনাকে কি কেউ ২/৩ টি (নাম) ট্যাবলেট দিয়েছিল?	হ্যাঁ......1<br>না......2	 → 503
502	ডেলিভারির পর পরই আপনি কি সেই ট্যাবলেটগুলো খেয়েছিলেন?	হ্যাঁ......1<br>না......2	
503	ডেলিভারির পর আপনার স্বাস্থ্য পরীক্ষা করার জন্য আপনি কি কোন স্বাস্থ্যকর্মীর কাছে গিয়েছিলেন?	হ্যাঁ......1<br>না......2	 → 507
504	আপনার স্বাস্থ্য পরীক্ষা করার জন্য কোন্ কোন্ স্বাস্থ্যকর্মীর কাছে আপনি গিয়েছিলেন?<br><br>**জিজ্ঞেস করুননঃ আরও কারও কাছে গিয়েছিলেন কি?**<br><br>**সব উত্তরের কোড বৃত্তায়িত করুন।**<br><br>**একাধিক উত্তর হতে পারে।**	পাশ করা (MBBS) ডাক্তার......A<br>নার্স/ধাত্রী......B<br>প্যারামেডিক......C<br>পরিবার কল্যাণ পরিদর্শিকা (FWV)......D<br>কম্যুউনিটি স্কিল্ড বার্থ এটেন্টডেন্ট (CSBA)......E<br>উপসহকারী কমিউনিটি চিকিৎসা কর্মকর্তা(সাকমো)......F<br>মা-মনি স্বাস্থ্যকর্মী/ CHW......G<br>স্বাস্থ্য সহকারী (HA)......H<br>পরিবার কল্যাণ সহকারী (FWA)......I<br>কমিউনিটি হেলথ কেয়ার প্রোভাইডার (CHCP)......J<br>প্রশিক্ষণপ্রাপ্ত টিবিএ (TTBA)......K<br>প্রশিক্ষণহীন টিবিএ (ধন্নী, চাউনি, দাই)......L<br>হোমিওপ্যাথ......M<br>আয়ুর্বেদিক চিকিৎসক......N<br>হাতুরে ডাক্তার/কোয়াক......O<br>গ্রাম ডাক্তার/পল-ী চিকিৎসক......P<br>ওঝা/ কবিরাজ......Q<br>অন্যান্য স্বাস্থ্যকর্মী......R<br>অন্যান্য__________ ......X<br>(নির্দিষ্ট করুন).<br>জানি না/মনে নাই......Y	
505	ডেলিভারির পর আপনার নিজের স্বাস্থ্য পরীক্ষা করার জন্য মোট কতবার আপনি স্বাস্থ্যকর্মীর কাছে গিয়েছিলেন?	বার...... [ ][ ]	
506	ডেলিভারির কতদিন পর প্রথমবার এবং দ্বিতীয়বার আপনার স্বাস্থ্য পরীক্ষা করার জন্য কোন স্বাস্থ্যকর্মীর কাছে গিয়েছিলেন?<br>**সাক্ষাতকারগ্রহণকারীঃ যদি একবার গিয়ে থাকে সেক্ষেত্রে দ্বিতীয়বার এর ঘরে "99" লিখুন।**	দিন পর<br>প্রথমবার...... [ ][ ]<br>দ্বিতীয়বার ...... [ ][ ]	

NO.	QUESTIONS AND FILTERS	CODING CATEGORIES	SKIP
	**এখন আমি আপনাকে আপনার ডেলিভারির পরের কিছু শারীরিক সমস্যার কথা জিজ্ঞেস করব।**		
507	ডেলিভারির পর আপনার কি ________হয়েছিল? (সমস্যা/জটিলতা)<br>**(প্রত্যেকটি সমস্যা/জটিলতা সম্পর্কে জিজ্ঞেস করুন)**	507a. **507 প্রশ্নে যে যে সমস্যার কোড 1 বৃত্তায়িত হবে, 507a প্রশ্নে সেই সমস্যা সম্পর্কেই জিজ্ঞেস করুন।**<br>**________ এর জন্য মোট কত দিন অসুস্থ ছিলেন?**<br>(সমস্যা/জটিলতা)<br>(১ দিনের কম হলে 00 লিখুন।)	

	সমস্যা/জটিলতা	হ্যাঁ	না	507a	দিন
a	জ্বর......................................	1 →	2	জ্বর......................................	__ __
B	যোনীপথে অতিরিক্ত রক্তস্রাব, বড় বড় রক্তের চাকা যাওয়া (রক্তস্রাব কমার চেয়ে বাড়া)	1 →	2	যোনীপথে অতিরিক্ত রক্তস্রাব, বড় বড় রক্তের চাকা যাওয়া (রক্তস্রাব কমার চেয়ে বাড়া)	__ __
C	যোনীপথে দুর্গন্ধযুক্ত স্রাব	1 →	2	যোনীপথে দুর্গন্ধযুক্ত স্রাব	__ __
d	তলপেটে তীব্র ব্যথা....................	1 →	2	তলপেটে তীব্র ব্যথা....................	__ __
e	শ্বাস নিতে কষ্ট,ক্লান্তি/অবসন্নতা, বুক ধরফরানি এবং দুর্বলতা....................	1 →	2	শ্বাস নিতে কষ্ট,ক্লান্তি/অবসন্নতা, বুক ধরফরানি এবং দুর্বলতা....................	__ __
f	নিস্তেজ/অজ্ঞানভাব....................	1 →	2	নিস্তেজ/অজ্ঞানভাব....................	__ __
g	ফিট/খিঁচুনী....................	1 →	2	ফিট/খিঁচুনী....................	__ __
h	মাসিকের রাস্তা দিয়ে অনবরত ফোঁটায় ফোঁটায় প্রস্রাব ঝরা....................	1 →	2	মাসিকের রাস্তা দিয়ে অনবরত ফোঁটায় ফোঁটায় প্রস্রাব ঝরা....................	__ __
i	স্তনে ব্যথা, প্রদাহ বা চাকা অথবা পূঁজ নির্গত হওয়া....................	1 →	2	স্তনে ব্যথা, প্রদাহ বা চাকা অথবা পূঁজ নির্গত হওয়া....................	__ __
j	অন্য কোন সমস্যা ________ (নির্দিষ্ট করুন)............	1 →	2	অন্য কোন সমস্যা ________ (নির্দিষ্ট করুন)........	__ __

NO.	QUESTIONS AND FILTERS	CODING CATEGORIES	SKIP
**508**	**সাক্ষাৎকারগ্রহণকারীঃ 507 দেখুন এবং সঠিক কোড বৃত্তায়িত করুন।**	**এক বা একাধিক কোড 1 বৃত্তায়িত....................1**<br>**সবগুলো কোড 2 বৃত্তায়িত..............................2**	**2 → 601**
509	________এর জন্য আপনি কি কোন চিকিৎসা (507 এর উত্তর) করিয়েছেন?	হ্যাঁ ....1<br>না...2<br>জানি না/মনে নেই...................................7	2, 7 → **601**
510	এই চিকিৎসা আপনি কোন্ কোন্ স্বাস্থ্যকর্মীর কাছে করিয়েছেন?<br><br>**জিজ্ঞেস করুনঃ আরও কার কাছে করিয়েছিলেন?**<br><br>**সব উত্তরের কোড বৃত্তায়িত করুন।**	পাশ করা (MBBS) ডাক্তার..............................A<br>নার্স/ধাত্রী ....B<br>প্যারামেডিক..C<br>পরিবার কল্যাণ পরিদর্শিকা (FWV).......................D<br>কম্যুউনিটি স্কিল্ড বার্থ এটেন্টডেন্ট (CSBA) .... E<br>উপসহকারী কমিউনিটি চিকিৎসা কর্মকর্তা(সাকমো) .... F<br>মা-মনি স্বাস্থ্যকর্মী/ CHW.................................G<br>স্বাস্থ্য সহকারী (HA)....................................... H<br>পরিবার কল্যাণ সহকারী (FWA).......................... I<br>কমিউনিটি হেলথ কেয়ার প্রোভাইডার (CHCP)..........J<br>প্রশিক্ষণপ্রাপ্ত টিবিএ (TTBA)...........................K<br>প্রশিক্ষণহীন টিবিএ (ধন্নী, চাউনি, দাই).................... L<br>হোমিওপ্যাথ. ....M<br>আয়ুর্বেদিক চিকিৎসক..N<br>হাতুরে ডাক্তার/কোয়াক ....O<br>গ্রাম ডাক্তার/পল্লী চিকিৎসক .... P<br>ওঝা/ কবিরাজ ....Q<br>অন্যান্য স্বাস্থ্যকর্মী..R<br>অন্যান্য________ (নির্দিষ্ট করুন). ....X<br>জানি না/মনে নাই...Y	

Section F: Newborn Care and Care Seeking

ডেলিভারির পর পরই বাচ্চার জন্য কিছু করণীয় থাকে। সে সম্পর্কে সুনির্দিষ্ট কিছু প্রশ্ন এখন আমি আপনাকে জিজ্ঞেস করব। অর্থাৎ (নাম) এর জন্মের পর পরই (নাম) কে কি করা হয়েছিল সে সম্পর্কে এখন আমি আপনাকে কিছু প্রশ্ন জিজ্ঞেস করব।

NO.	QUESTIONS AND FILTERS	CODING CATEGORIES	SKIP
**601**	**সাক্ষাৎকারগ্রহনকারীঃ প্রশ্ন 408 দেখুন এবং সঠিক কোড বৃত্তায়িত করুন।**	**কোড 11 বা 12 বা 13 বা 43 বা 96 বৃত্তায়িত............. 1**<br>**কোড 21 থেকে কোড 42 এর যে কোন একটি বৃত্তায়িত 2**	 **→611**
602	_____এর জন্মের সাথে সাথে অর্থাৎ পেট থেকে বের<br>(নাম)<br>হওয়ার পর পরই কোন্ কাজটি প্রথম করা হয়েছিল?<br><br>**(প্রোব করুন।)**<br><br>**শুধুমাত্র একটি উত্তর বৃত্তায়িত করুন।**	নাড়ী কাটা .... 01<br>মায়ের পেটের/বুকের উপর বাচ্চাকে রাখা........ 02<br>একা ফেলে রাখা .... 03<br>গা শুকানো .... 04<br>কাপড় দিয়ে মুড়ানো .... 05<br>গোসল করানো .... 06<br>বাচ্চাকে ঘুমাতে দেয়া........ 07<br>বুকের দুধ খাওয়ানো........ 08<br>চিনির পানি বা অন্য কিছু খাওয়ানো........ 09<br>অন্যান্য _______________ .... 96<br>**(নির্দিষ্ট করুন)**<br>জানিনা/মনে নাই 97	
603	_____ এর জন্মের কতক্ষণ পর ফুল পড়েছিল?<br>(নাম)	মিনিট .... ☐☐<br>**বাচ্চার সাথেই ফুল পড়েছিল........** 94<br>ফুল পড়ে নি, আমাকে হাসপাতালে নিয়ে যাওয়া হয়েছিল .... 95<br>জানি না........ 97	
604	_____কে কখন মোছানো/শুকানো হয়েছিল, ফুল<br>(নাম)<br>পড়ার আগে না-কি ফুল পড়ার পরে?	ফুল পড়ার আগে .... 1<br>ফুল পড়ার পর........ 2<br>মোছানো/শুকানো হয় নি........ 3<br>জানি না/মনে নেই........ 7	
605	জন্মের পর কখন _____ কে কাপড় দিয়ে মুড়িয়ে<br>(নাম)<br>নেয়া হয়েছিল, ফুল পড়ার আগে না-কি ফুল পড়ার পরে?	ফুল পড়ার আগে .... 1<br>ফুল পড়ার পড়ে .... 2<br>কাপড় দিয়ে মুড়ানো হয় নি .... 3<br>জানিনা/মনে নেই........ 7	
606	_____ এর নাড়ী কি দিয়ে কাটা হয়েছিল?<br>(নাম)	ডেলিভারি ব্যাগ এর বে- ড .... 01<br>নতুন বে- ড........ 02<br>বাড়ীর পুরাতন বে- ড .... 03<br>বাঁশের কঞ্চি/বাতা/টিন .... 04<br>কাঁচি........ 05<br>অন্যান্য _______________ .... 96<br>**(নির্দিষ্ট করুন)**<br>জানি না/মনে নেই........ 97	 <br><br><br><br><br><br>**→609**
607	নাড়ী কাটার আগে________টি সিদ্ধ করে/ পানিতে<br>(606 এর উত্তর)<br>ফুটিয়ে নেয়া হয়েছিল কি?	হ্যাঁ .... 1<br>না .... 2<br>জানিনা/মনে নেই........ 7	
608	জন্মের কতক্ষণ পর _____এর নাড়ী কাটা এবং<br>(নাম)<br>বাঁধা হয়েছিল?	মিনিট........ ☐☐<br>জানিনা 97	
609	নাড়ী কাটা এবং বাঁধার পর পরই তাতে কিছু দেয়া হয়েছিল কি?	হ্যাঁ........ 1<br>না........ 2<br>জানিনা/মনে নেই 7	 **→ 611** (2, 7)

NO.	QUESTIONS AND FILTERS	CODING CATEGORIES	SKIP
610	নাড়ী কাটা এবং বাঁধার পর পরই তাতে কি দেয়া হয়েছিল?<br><br>**জিজ্ঞেস করুনঃ আরও কিছু দেয়া হয়েছিল কি?**<br><br>**সব উত্তরের কোড বৃত্তায়িত করুন।**<br><br>**উত্তর একাধিক হতে পারে।**	অ্যান্টিবায়োটিক (পাউডার/মলম)______________ ....A<br>(নির্দিষ্ট করুন)<br>অ্যান্টিসেপটিক (ডেটল/স্যাভলন/হেক্সাসল) ....B<br>স্পিরিট/অ্যালকোহল...C<br>সরিষার তেল (রসুন সহ বা বাদে)................................ D<br>চিবানো চাল...E<br>হলুদের রস/গুড়া.. F<br>আদার রস.. G<br>সিঁদুর.. H<br>বরিক পাউডার ....I<br>জেনসিয়ন ভায়োলেট/নীল কালি ....J<br>ট্যালকম পাউডার.. K<br>ছাই..L<br>নারিকেল তেল ....M<br>চুলার মাটি.. N<br>অন্যান্য ______________.................................. X<br>**(নির্দিষ্ট করুন)**<br>জানিনা.. Y	
611	নাড়ী কাটা এবং বাঁধার ৭ দিনের মধ্যে নাড়ীতে কিছু দিয়েছিলেন কি?	হ্যাঁ...1<br>না.. 2<br>জানিনা...7	2, 7 → **613**
612	নাড়ী কাটা এবং বাঁধার ৭ দিনের মধ্যে নাড়ীতে কি দেয়া হয়েছিল?<br><br>**জিজ্ঞেস করুনঃ আরও কিছু দেয়া হয়েছিল কি?**<br><br>**সব উত্তরের কোড বৃত্তায়িত করুন।**<br><br>**উত্তর একাধিক হতে পারে।**	অ্যান্টিবায়োটিক (পাউডার/মলম)_________ .... A<br>(নির্দিষ্ট করুন)<br>অ্যান্টিসেপটিক (ডেটল/স্যাভলন/হেক্সাসল) ....B<br>স্পিরিট/অ্যালকোহল...C<br>সরিষার তেল (রসুন সহ বা বাদে)................................ D<br>চিবানো চাল...E<br>হলুদের রস/গুড়া.. F<br>আদার রস.. G<br>সিঁদুর.. H<br>বরিক পাউডার ....I<br>জেনসিয়ান ভায়োলেট/নীল কালি......................................J<br>ট্যালকম পাউডার.. K<br>ছাই..L<br>নারিকেল তেল ....M<br>চুলার মাটি.. N<br>অন্যান্য ______________.................................. X<br>**(নির্দিষ্ট করুন)**<br>জানিনা.. Y	
613	আপনার ডেলিভারির প্রথম ৭ দিনের মধ্যে _____কে<br>(নাম)<br>দেখার জন্য মা-মনি/কমিউনিটি স্বাস্থ্যকর্মী এসেছিলেন কি?	হ্যাঁ ....1<br>না...2<br>জানি না 7	2, 7 → **615**
614	ডেলিভারির ৭ দিনের মধ্যে মা-মনি স্বাস্থ্যকর্মী মোট কতবার এসেছিলেন?	বার .... ☐☐	
615	জন্মের পর পরই _____স্বাভাবিকভাবে/নরমালি<br>(নাম)<br>কেঁদেছিল/শ্বাস নিয়েছিল কি?	হ্যাঁ.. 1<br>না.. 2<br>জানিনা/মনে নেই.. 7	1 → **618**
616	জন্মের পর পরই _____ কে কাঁদানোর জন্য বা শ্বাস<br>(নাম)<br>নেয়ানোর জন্য কিছু করতে হয়েছিল কি?	হ্যাঁ... 1<br>না.. 2<br>জানিনা..7	2, 7 → **618**
616a	জন্মের পর পরই _____ কে কাঁদানোর জন্য বা শ্বাস<br>(নাম)<br>নেয়ানোর জন্য কি করতে হয়েছিল?<br><br>**জিজ্ঞেস করুনঃ আরও কিছু ?**<br><br>**উত্তর একাধিক হতে পারে।**	বাচ্চার পিঠে ঘষা দিয়ে উত্তেজিত করা হয়েছে .... A<br>বাচ্চার পায়ের পাতা ঘষা দিয়ে উত্তেজিত করা হয়েছে ....B<br>মুখ থেকে মুখে শ্বাস নেয়ানোর চেষ্টা করা হয়েছে.................C<br>নাড়ীতে তাপ দেয়া হয়েছে.. D<br>বাচ্চাকে থাপ্পড় দেয়া হয়েছে ....E<br>বাচ্চার মাথা নিচ দিকে দিয়ে ঝুলানো হয়েছে .... F<br>অন্যান্য______________ ....X<br>(নির্দিষ্ট করুন)<br>জানিনা/মনে নেই .... Y	Y → **618**

NO.	QUESTIONS AND FILTERS	CODING CATEGORIES	SKIP
617	_____কে কাঁদানোর/শ্বাস নেয়ানোর জন্য কে চেষ্টা বা<br>(নাম)<br>কিছু করেছিলেন?<br><br>**উত্তর একাধিক হতে পারে।**	পাশ করা (MBBS) ডাক্তার............................ A<br>নার্স/ধাত্রী ....B<br>প্যারামেডিক ....C<br>পরিবার কল্যাণ পরিদর্শিকা .... D<br>কম্যুউনিটি স্কিল্ড বার্থ এটেন্টডেন্ট (CSBA)..............E<br>উপসহকারী কমিউনিটি চিকিৎসা কর্মকর্তা(সাকমো) .... F<br>মা-মনি স্বাস্থ্যকর্মী/ কমিউনিটি স্বাস্থ্যকর্মী .... G<br>স্বাস্থ্য সহকারী .... H<br>পরিবার কল্যাণ সহকারী ....I<br>প্রশিক্ষণপ্রাপ্ত টিবিএ..J<br>প্রশিক্ষণহীন টিবিএ (ধন্নী, চাউনি, দাই).................... K<br>হোমিওপ্যাথ ....L<br>আয়ুর্বেদিক চিকিৎসক..M<br>হাতুড়ে ডাক্তার/কোয়াক .... N<br>গ্রাম ডাক্তার/পল-ী চিকিৎসক .... O<br>ওঝা/ কবিরাজ..P<br>মা/শ্বাশুড়ী .... Q<br>পরিবারের সদস্য/আত্মীয় ....R<br>প্রতিবেশী/বন্ধু...S<br>মা নিজেই...T<br>অন্যান্য ______________ .... X<br>(নির্দিষ্ট করু্ন)<br>জানিনা/মনে নাই.. Y	
618	_____ জন্মের পর আকারে কতটুকু ছিল?<br>(নাম)<br>স্বাভাবিকের চেয়ে অনেক ছোট, নাকি স্বাভাবিকের থেকে একটু ছোট, নাকি স্বাভাবিক, নাকি স্বাভাবিকের চেয়ে বড়?	অনেক ছোট .... 1<br>স্বাভাবিকের থেকে ছোট .... 2<br>স্বাভাবিক.. 3<br>স্বাভাবিকের চেয়ে বড় .... 4<br>ছোট না বড়, বুঝি নাই....................................... 7	
619	জন্মের পর _____ এর ওজন কত ছিল?<br>(নাম)<br>**সাক্ষাৎকারগ্রহনকারীঃ কার্ড দেখাতে পারলে, কার্ড থেকে ওজন লিখুন।**<br><br>**কার্ড না দেখাতে পারলে, শুনে ওজন লিখুন।**	জন্ম ওজন<br>কেজি.............................1 [ ].[ ][ ]<br>পাউন্ড ....2 [ ].[ ][ ]<br>ওজন নেয়া হয় নাই .... 9995<br>জানি না/মনে নাই....................................... 9997	
620	_____ কি নির্দিষ্ট সময় (৩৬ সপ্তাহ) এর আগে<br>(নাম)<br>জন্ম নিয়েছিল?	হ্যাঁ.. 1<br>না... 2<br>জানিনা/মনে নেই 7	
621	_____ কে জন্মের পর কখন প্রথম গোসল করানো<br>(নাম)<br>হয়েছিল?<br>**সাক্ষাৎকারগ্রহনকারীঃ যদি জন্মের 24 ঘন্টার মধ্যে গোসল করানো হয় তাহলে ঘন্টায় লিখুন। 24 ঘন্টার পরে গোসল করানো হলে দিনে লিখুন।**	সাথে সাথেই ....000<br>ঘন্টা..1 [ ][ ]<br>দিন...2 [ ][ ]<br>গোসল করানো হয় নাই ....996<br>জানিনা..997	

NO.	QUESTIONS AND FILTERS	CODING CATEGORIES	SKIP
622	জন্মের পর পর _____এর শরীর গরম রাখার জন্য কি<br>(নাম)<br>করেছিলেন?<br><br>**জিজ্ঞেস করুনঃ আরও কিছু?**<br><br>**সব উত্তরের কোড বৃত্তায়িত করুন।**<br><br>**উত্তর একাধিক হতে পারে।**	শুকিয়েছিলাম ....A<br>পরিস্কার কাপড়/কাঁথা দিয়ে মুড়িয়ে নিয়েছিলাম.............B<br>বুকের চামড়ার উপরে বাচ্চাকে চেপে রেখেছিলাম ....C<br>রান্নাঘরে ডেলিভারি করানো হয়েছিল ....D<br>ডেলিভারি কক্ষে আগুন জ্বালিয়ে রাখার<br>ব্যবস্থা করা হয়েছিল..E<br>গরম তেল শরীরে মালিশ করেছিলাম.........................F<br>আমার কোলে বাচ্চাকে রেখেছিলাম ....G<br>অন্য কারো কোলে বাচ্চাকে রাখা হয়েছিল ....H<br>ইনকিউবেটরে রাখা হয়েছিল....................................I<br>অন্যান্য _______________..............................X<br>(নির্দিষ্ট করুন)<br>জানি না ....Y<br>কিছুই করি নি..Z	
623	জন্মের ১৫ দিনের মধ্যে দিন এবং রাতের বেলায় প্রতিদিন কত ঘন ঘন _____ র খালি বুক আপনার বুকের মধ্যে<br>(নাম)<br>(খালি বুকে) রেখেছিলেন?	সারাক্ষণ.. 1<br>প্রায়ই সারাক্ষণ.. 2<br>প্রায়ই.. 3<br>খুব কম.. 4<br>কখনই নয়... 5<br>জন্মের পর পর বাচ্চা মারা গিয়েছে........................... 6	5, 6 → **625**
624	জন্মের ১৫ দিনের মধ্যে রাতে ঘুমানোর সময় _____কে<br>(নাম)<br>বুকে রাখতেন না-কি একই বিছানায় না আলাদা রাখতেন?	বাচ্চাকে বুকে রাখতাম .... 1<br>বাচ্চাকে একই বিছানায় রাখতাম .... 2<br>বাচ্চাকে বুকে রাখতাম এবং বিছানায়ও রাখতাম........... 3<br>আলাদা রাখতাম 4	
625	_____ কে কখনও বুকের দুধ খাইয়েছিলেন কি?<br>(নাম)	হ্যাঁ .... 1<br>না 2	2 → **629**
626	ফুল পড়া বা বের হওয়ার আগেই কি_____ কে বুকের<br>(নাম)<br>দুধ খাওয়ানো হয়েছিল?	হ্যাঁ... 1<br>না .... 2<br>জানিনা/মনে নেই 7	
627	জন্মের কত সময় পর _____ কে প্রথম বুকের দুধ<br>(নাম)<br>দিতে শুরু করেছিলেন?<br>**সাক্ষাৎকারগ্রহনকারীঃ জন্মের 1 ঘন্টার মধ্যে হলে 00 বৃত্তায়িত করুন। 24 ঘন্টার মধ্যে হলে ঘন্টায় লিখুন। 24 ঘন্টা বা তার অধিক হলে উত্তর দিনে লিখুন।**	জন্মের পর পর...000<br>ঘন্টা ....1 [ ][ ]<br>দিন...2 [ ][ ]	
628	_____ এর জন্মের প্রথম তিন দিনের মধ্যে বুকের দুধ<br>(নাম)<br>ছাড়া অন্য কিছু খাইয়েছিলেন কি?	হ্যাঁ.. 1<br>না.. 2<br>জানিনা/মনে নেই 7	2, 7 → **630**
629	_____ কে জন্মের প্রথম তিন দিনের মধ্যে কি কি<br>(নাম)<br>খাইয়েছিলেন?<br><br>**জিজ্ঞেস করুনঃ আরও কিছু খাইয়েছিলেন কি?**<br><br>**সব উত্তরের কোড বৃত্তায়িত করুন।**<br><br>**উত্তর একাধিক হতে পারে।**	মধু...A<br>মিস্রীর পানি..B<br>চিনির পানি ....C<br>পানি...D<br>ফলের রস ....E<br>টিনজাত দুধ/শিশু খাদ্য (বেবী ফর্মুলা) ....F<br>গরুর/**ছাগলের** দুধ..G<br>অন্যান্য তরল..H<br>লেই (পানিতে মিশানো চালের গুড়া, আটা, ময়দা)............I<br>অন্যান্য_______________...................................X<br>(নির্দিষ্ট করুন)<br>**কিছু খাওয়াই নি**...Z	

NO.	QUESTIONS AND FILTERS	CODING CATEGORIES	SKIP
630	জন্মের কতক্ষণের/কতদিনের মধ্যে ____ প্রস্রাব করেছিল? (নাম)<br>**১ দিনের কম হলে ঘন্টায় লিখুন।**	জন্মের পর পর..000<br>ঘন্টা .... 1 ☐☐<br>দিন .... 2 ☐☐<br>জানি না/মনে নেই 997	
630a	জন্মের কতক্ষণের/কতদিনের মধ্যে _____পায়খানা (নাম) করেছিল?<br>**১ দিনের কম হলে ঘন্টায় লিখুন।**	জন্মের পর পর..000<br>ঘন্টা .... 1 ☐☐<br>দিন .... 2 ☐☐<br>জানি না/মনে নেই 997	
631	_____এর জন্মের পর জন্ম নিবন্ধন করিয়েছিলেন? (নাম)	হ্যাঁ.. 1<br>না.. 2<br>জানিনা/মনে নেই 7	1→ **633**
632	কেন _____ এর জন্মের পর জন্ম নিবন্ধন করানো হয় (নাম) নাই?<br><br>**জিজ্ঞেস কর‍ুনঃ আরও কিছু?**<br><br>**সব উত্তরের কোড বৃত্তায়িত কর‍ুন।**<br><br>**উত্তর একাধিক হতে পারে।**	পরিকল্পনা আছে, ভবিষ্যতে করব ....A<br>জন্ম নিবন্ধন কি সেটা জানি না ....B<br>জানতাম না কোথায় করাতে হবে .... C<br>জন্ম নিবন্ধনের প্রয়োজন জানি না.......................... D<br>জন্ম নিবন্ধনের প্রয়োজন বোধ করি নাই.................... E<br>ইউনিয়ন পরিষদ/ পৌরসভা বাসা হতে অনেক দুরে....... F<br>সাথে যাবার মত কেউ ছিল না............................. G<br>ইউনিয়ন পরিষদ/পৌরসভা যাবার মত সময় ছিল না..... H<br>**বাচ্চা মারা গিয়েছে**..I<br>অন্যান্য____________________ ....X<br>(নির্দিষ্ট কর‍ুন)	
**এবার আমরা আপনার কাছে (নাম) এর জন্মের পরের স্বাস্থ্য পরীক্ষা সংক্রান্ত কিছু প্রশ্ন করবো।**			
633	_____ এর জন্মের পর ওর স্বাস্থ্য পরীক্ষা করার জন্য (নাম) আপনি কোন স্বাস্থ্যকর্মীর কাছে গিয়েছিলেন কি?	হ্যাঁ ....1<br>না 2	2→ **636**
634	_____এর জন্মের কত দিন/মাস পর স্বাস্থ্য পরীক্ষা (নাম) করার জন্য আপনি স্বাস্থ্যকর্মীর কাছে গিয়েছিলেন?<br>**সাক্ষাৎকারগ্রহনকারীঃ ১ দিনের কম হলে 00 লিখুন। ১ মাসের কম হলে দিনে, ১ মাসের বেশী হলে মাসে লিখুন।**	দিন.. 1 ☐☐<br>মাস .... 2 ☐☐	
635	_____এর স্বাস্থ্য পরীক্ষা করার জন্য কোন কোন (নাম) স্বাস্থ্যকর্মীর কাছে আপনি গিয়েছিলেন?<br><br>**জিজ্ঞেস কর‍ুনঃ আরও কারও কাছে গিয়েছিলেন কি?**<br>**সব উত্তরের কোড বৃত্তায়িত কর‍ুন।**<br>**উত্তর একাধিক হতে পারে।**	পাশ করা (MBBS) ডাক্তার..............................A<br>নার্স/ধাত্রী ....B<br>প্যারামেডিক ....C<br>পরিবার কল্যাণ পরিদর্শিকা (FWV).......................D<br>কম্যুউনিটি স্কিল্ড বার্থ এটেন্টডেন্ট (CSBA).............E<br>উপসহকারী কমিউনিটি চিকিৎসা কর্মকর্তা(সাকমো) ....F<br>মা-মনি স্বাস্থ্যকর্মী/ কমিউনিটি স্বাস্থ্যকর্মী ....G<br>স্বাস্থ্য সহকারী ....H<br>পরিবার কল্যাণ সহকারী (FWA)...........................I<br>প্রশিক্ষণপ্রাপ্ত টিবিএ .... J<br>প্রশিক্ষণহীণ টিবিএ (ধন্নী, চাউনি, দাই)....................K<br>হোমিওপ্যাথ .... L<br>আয়ুর্বেদিক চিকিৎসক.. M<br>হাতুরে ডাক্তার/কোয়াক..N<br>গ্রাম ডাক্তার/পল-ী চিকিৎসক ....O<br>ওঝা/কবিরাজ..P<br>অন্যান্য স্বাস্থ্যকর্মী..Q<br>অন্যান্য ____________________......................X<br>**(নির্দিষ্ট কর‍ুন)**<br>জানি না/মনে নাই..Y	

NO.	QUESTIONS AND FILTERS	CODING CATEGORIES		SKIP
	**এখন আমি আপনার বাচ্চার স্বাস্থ্য সমস্যা সম্পর্কে কিছু প্রশ্ন জিজ্ঞেস করতে চাই।**			
636	জন্মের ১ মাসের মধ্যে _____কি কোন ধরনের (নাম) অসুস্থতায় ভুগেছে?	হ্যাঁ.......1<br>না.......2<br>জানিনা/মনে নেই 7		2, 7 → **701**
637	জন্মের ১ মাসের মধ্যে _____ কি কি ধরনের (নাম) অসুস্থতায় ভুগেছে?<br>**জিজ্ঞেস করুনঃ আরও কিছু ?**	637a.	**637 প্রশ্নে যে কোড বৃত্তায়িত হবে, 637a প্রশ্নে সেটি সম্পর্কে জিজ্ঞেস করুন।**<br>_________ এর জন্য____ মোট কত দিন অসুস্থ ছিল? (সমস্যা/অসুবিধা) (নাম)<br>(১ দিনের কম হলে 00 লিখুন।) **দিন**	
	কষ্টকর/দ্রুত শ্বাস নেয়া.......A		কষ্টকর/দ্রুত শ্বাস নেয়া....... [ ][ ]	
	বুকের খাঁচা ডেবে যাওয়া.......B		বুকের খাঁচা ডেবে যাওয়া....... [ ][ ]	
	নিউমোনিয়া.......C		নিউমোনিয়া....... [ ][ ]	
	খিঁচুনী/শরীর শক্ত.......D		খিঁচুনী/শরীর শক্ত....... [ ][ ]	
	বাচ্চার শরীর ঠান্ডা হওয়া.......E		বাচ্চার শরীর ঠান্ডা হওয়া....... [ ][ ]	
	বাচ্চার খাওয়া কমে যাওয়া/বুকের দুধ চুষতে না পারা . F		বাচ্চার খাওয়া কমে যাওয়া/বুকের দুধ চুষতে না পারা....... [ ][ ]	
	নাভির চারপাশে লাল হওয়া/কিছু বের হওয়া.......G		নাভির চারপাশে লাল হওয়া/কিছু বের হওয়া....... [ ][ ]	
	ঘুম থেকে জাগানো কষ্টকর.......H		ঘুম থেকে জাগানো কষ্টকর....... [ ][ ]	
	ঠান্ডা/কফ/সর্দি/কাশি....... I		ঠান্ডা/কফ/সর্দি/কাশি....... [ ][ ]	
	অচেতন/অজ্ঞান/হুঁশ না থাকা.......J		অচেতন/অজ্ঞান/হুঁশ না থাকা....... [ ][ ]	
	চোখ লাল হওয়া/ময়লা বা পিঙ্গিস বের হওয়া......K		চোখ লাল হওয়া/ময়লা বা পিঙ্গিস বের হওয়া....... [ ][ ]	
	চামড়ার রং, হাত, হাতের তালু, পায়ের পাতা, চোখ হলুদ হওয়া/জন্ডিস/ওলমি....... L		চামড়ার রং, হাত, হাতের তালু, পায়ের পাতা,চোখ হলুদ হওয়া/জন্ডিস/ওলমি. [ ][ ]	
	বাচ্চা না কাঁদা.......M		বাচ্চা না কাঁদা....... [ ][ ]	
	জ্বর.......N		জ্বর....... [ ][ ]	
	প্রস্রাব না হওয়া.......O		প্রস্রাব না হওয়া....... [ ][ ]	
	পায়খানা না করা....... P		পায়খানা না করা....... [ ][ ]	
	একটানা বমি.......Q		একটানা বমি....... [ ][ ]	
	পেট ফাঁপা.......R		পেট ফাঁপা....... [ ][ ]	
	চামড়ায় ফোসকা/ঘা হওয়া....... S		চামড়ায় ফোসকা/ঘা হওয়া....... [ ][ ]	
	চামড়ায় ফুসকুড়ি/র‍্যাশ/মাসিপিসি....... T		চামড়ায় ফুসকুড়ি/র‍্যাশ/মাসিপিসি....... [ ][ ]	
	হাম / প্যারা / ফ্যারা.......U		হাম / প্যারা / ফ্যারা....... [ ][ ]	
	ধনুষ্টঙ্কার.......V		ধনুষ্টঙ্কার....... [ ][ ]	
	**অন্যান্য**_______________.......X (নির্দিষ্ট করুন)		**অন্যান্য**_______________ .... [ ][ ] (নির্দিষ্ট করুন)	
638	_____এর এই অসুস্থতার জন্য আপনি কোন চিকিৎসা (নাম) করিয়েছেন কি?	হ্যাঁ.......1<br>না ....2<br>জানিনা/মনে নেই 7		2, 7 → **659**
639	এই _________ সনাক্ত করার কতক্ষণ পর _____ এর (637 এর উত্তর) (নাম) চিকিৎসা করতে হবে সেই সিদ্ধান্ত নেয়া হয়েছিল?<br>**সাক্ষাৎকারগ্রহনকারীঃ ১ দিনের কম হলে ঘন্টায়, ১ দিন বা তার বেশি হলে পূর্ণ দিনে লিখুন**	সাথে সাথে.......000<br>ঘন্টা পর.......1 [ ][ ]<br>দিন পর ....2 [ ][ ]<br>জানি না.......997		

NO.	QUESTIONS AND FILTERS	CODING CATEGORIES	SKIP
640	এই ________ এর জন্য আপনি কার কার কাছে<br>(637 এর উত্তর)<br>_____এর চিকিৎসা করিয়েছেন?<br>(নাম)<br>**জিজ্ঞেস করুনঃ আরও কার কাছে গিয়েছিলেন?**<br>**সব উত্তরের কোড বৃত্তায়িত করুন।**<br>**উত্তর একাধিক হতে পারে।**	পাশ করা (MBBS) ডাক্তার ....A<br>নার্স/ধাত্রী..B<br>প্যারামেডিক ....C<br>পরিবার কল্যাণ পরিদর্শিকা (FWV) ....D<br>কম্যুউনিটি স্কিল্ড বার্থ এটেন্টডেন্ট (CSBA)......................... E<br>চিকিৎসা সহকারী/উপসহকারী কমিউনিটি চিকিৎসা কর্মকর্তা (সাকমো).. F<br>মা-মনি স্বাস্থ্যকর্মী/কমিউনিটি স্বাস্থ্যকর্মী ....G<br>স্বাস্থ্য সহকারী (HA)...H<br>পরিবার কল্যাণ সহকারী (FWA)............................ I<br>প্রশিক্ষণপ্রাপ্ত টিবিএ ....J<br>প্রশিক্ষণহীন টিবিএ (ধন্নী, চাউনি, দাই).......................K<br>হোমিওপ্যাথ...L<br>আয়ুর্বেদিক চিকিৎসক..M<br>হাতুড়ে ডাক্তার/কোয়াক...N<br>গ্রাম ডাক্তার/পল্লী চিকিৎসক................................O<br>ওঝা/কবিরাজ... P<br>অন্যান্য স্বাস্থ্যকর্মী ....Q<br>অন্যান্য ________________ ....X<br>(নির্দিষ্ট করুন)<br>জানিনা/মনে নেই...Y	
641.	____ এর চিকিৎসার জন্য মোট কতবার (ভর্তি ছাড়া এবং ভর্তিসহ)<br>(নাম)<br>ডাক্তারের কাছে/স্বাস্থ্যকেন্দ্রে/হাসপাতালে যেতে হয়েছিল?	বার.. [ ][ ]	
642	_____ এর অসুস্থতার জন্য স্বাস্থ্যকেন্দ্রে/ হাসপাতালে<br>(নাম)<br>ভর্তি হতে হয়েছিল কি?	হ্যাঁ .... 1<br>না 2	 → **646**
643	_____ কে কোন্ স্বাস্থ্যকেন্দ্রে/ হাসপাতালে ভর্তি হতে<br>(নাম)<br>হয়েছিল?<br>**উত্তর একাধিক হতে পারে।**	**সরকারী স্বাস্থ্যকেন্দ্র**<br>মেডিকেল কলেজ হাসপাতাল.....................................A<br>জেলা/সদর হাসপাতাল...B<br>মা ও শিশু স্বাস্থ্যকেন্দ্র...C<br>উপজেলা স্বাস্থ্য কমপ্লেক্স..D<br>ইউনিয়ন স্বাস্থ্য ও পরিবার কল্যাণ কেন্দ্র/ সাব সেন্টার/আরডি .... E<br>কমিউনিটি ক্লিনিক .... F<br>**এনজিও স্বাস্থ্যকেন্দ্র**<br>এনজিও হাসপাতাল..G<br>এনজিও স্থায়ী স্বাস্থ্যকেন্দ্র...H<br>প্রাইভেট/বেসরকারী প্রাইভেট হাসপাতাল/ ক্লিনিক .... I<br>অন্যান্য প্রাইভেট স্বাস্থ্যকেন্দ্র...J<br>অন্যান্য ________________ ....X<br>(নির্দিষ্ট করুন)	
644	______ এর অসুস্থতার জন্য স্বাস্থ্যকেন্দ্রে/হাসপাতালে<br>(নাম)<br>মোট কতবার ভর্তি হতে হয়েছিল?	বার... [ ]	
645	______ (প্রতিবার) কতদিন ভর্তি ছিল?<br>(নাম)	দিন<br>প্রথমবার .... [ ][ ]<br>দ্বিতীয়বার .... [ ][ ]<br>তৃতীয়বার .... [ ][ ]	

	**Expenditure related to Neonatal illness:**				
**646**	**সাক্ষাৎকারগ্রহনকারীঃ প্রশ্ন 641দেখুন। উত্তর একবার হলে শুধু মাত্র ১ম ভিজিটের কলাম, দুইবার হলে ১ম ও ২য় ভিজিটের কলাম, তিনবার হলে তিনটি কলামই 647 থেকে 655a পর্যন্ত প্রশ্নগুলো জিজ্ঞেস করুন। তিনবারের অধিক হলে অতিরিক্ত শীট ব্যবহার করুন।**				
	**_____এর চিকিৎসার জন্য আপনি মোট___ বার ডাক্তারের কাছে/হাসপাতালে গিয়েছিলেন। আমি এখন আপনার কাছ**<br>(নাম)<br>**থেকে প্রত্যেকবারের চিকিৎসার খরচ আলাদা আলাদা করে জানতে চাইব।**<br>**(সাক্ষাৎকারগ্রহনকারীঃ উত্তরদাতা বলতে না পারলে পরিবারের অন্যান্যদের সাহায্য নিন।)**				
		১ম ভিজিট	২য় ভিজিট	৩য় ভিজিট	
647	_____এর চিকিৎসার জন্যে যে ডাক্তারের<br>(নাম)<br>কাছে/স্বাস্থ্যকেন্দ্রে/হাসপাতালে গিয়েছিলেন আপনার বাড়ী থেকে এর দূরত্ব কত?	কি মি.... ☐☐<br>**স্বাস্থ্যকেন্দ্রে** জন্মের পর পরই অসুস্থ হয়েছিল... 95<br>**(653 এ যান)**	কি মি .... ☐☐	কি মি.. ☐☐	
648	ওই ডাক্তারের কাছে/স্বাস্থ্যকেন্দ্রে/হাসপাতালে আপনি কিসে গিয়েছিলেন ?<br>**সাক্ষাৎকারগ্রহনকারীঃ যদি একাধিক যানবাহন ব্যবহার করে থাকেন তাহলে সব থেকে বেশী দূরত্ব অতিক্রম করতে যে যানবাহন ব্যবহার করেছেন সে সম্পর্কে জিজ্ঞেস করুন।**	রিকশা/ ভ্যান ....01<br>বাস ....02<br>টেম্পু ....03<br>মটরসাইকেল ....04<br>সি এন জি ....05<br>এম্বুলেন্স ....06<br>নৌকা ....07<br>পায়ে হেঁটে ....08<br>অন্যান্য ______.....96<br>(নির্দিষ্ট করুন)	রিকশা/ ভ্যান...........01<br>বাস ....02<br>টেম্পু ....03<br>মটরসাইকেল ....04<br>সি এন জি ....05<br>এম্বুলেন্স ....06<br>নৌকা ....07<br>পায়ে হেঁটে ....08<br>অন্যান্য ______ ......96<br>(নির্দিষ্ট করুন)	রিকশা/ ভ্যান ....01<br>বাস ....02<br>টেম্পু ....03<br>মটরসাইকেল ......04<br>সি এন জি ....05<br>এম্বুলেন্স ....06<br>নৌকা ....07<br>পায়ে হেঁটে ....08<br>অন্যান্য ______...96<br>(নির্দিষ্ট করুন)	
649	ওই ডাক্তারের কাছে/স্বাস্থ্যকেন্দ্রে/হাসপাতালে যেতে কত সময় লেগেছিল? (যাওয়ার মোট সময় এবং যানবাহনের জন্য অপেক্ষারত সময় সহ বলবেন।)<br>**১ ঘন্টার কম হলে মিনিটে লিখুন।**<br>**১ ঘন্টার বেশি হলে পূর্ণ ঘন্টায় লিখুন।**	মিনিট .1 ☐☐<br>ঘন্টা ..2 ☐☐<br>জানিনা............ 997	মিনিট. 1 ☐☐<br>ঘন্টা . 2 ☐☐<br>জানিনা............ 997	মিনিট .1 ☐☐<br>ঘন্টা ..2 ☐☐<br>জানিনা 997	
650	ওই ডাক্তারের কাছে/স্বাস্থ্যকেন্দ্রে / হাসপাতালে যেতে আপনার মোট কত টাকা খরচ হয়েছিল?<br>**(কোন খরচ না হলে ”0000 লিখুন; জানিনা হলে পুণরায় জিজ্ঞাসা করুন যে পরিবারের কেউ জানে কিনা, কেউ জানলে তার কাছ থেকে শুনে লিখুন, না হলে 9997 লিখুন)**	টাকা☐☐☐☐	টাকা ☐☐☐☐	টাকা ☐☐☐☐	
651	ওই ডাক্তারের কাছে/স্বাস্থ্যকেন্দ্রে / হাসপাতাল থেকে ফেরার সময় আপনার মোট কত খরচ হয়েছিল? **(কোন খরচ না হলে 0000 লিখুন; জানিনা হলে পুণরায় জিজ্ঞাসা করুন যে পরিবারের কেউ জানে কিনা, কেউ জানলে তার কাছ থেকে শুনে লিখুন, না হলে 9997 লিখুন)**	টাকা☐☐☐☐	টাকা ☐☐☐☐	টাকা ☐☐☐☐	
652	ডাক্তারের কাছে/স্বাস্থ্যকেন্দ্রে/হাসপাতালে পৌছাবার পর থেকে চিকিৎসা পাবার আগ পর্যন্ত আপনাকে মোট কত সময় অপেক্ষা করতে হয়েছিল?<br>১ ঘন্টার কম হলে মিনিটে লিখুন। ১ ঘন্টার বেশী হলে পূর্ণ ঘন্টায় লিখুন।	মিনিট .1 ☐☐<br>ঘন্টা ..2 ☐☐<br>জানিনা......... 997	মিনিট. 1 ☐☐<br>ঘন্টা . 2 ☐☐<br>জানিনা......... 997	মিনিট .1 ☐☐<br>ঘন্টা ..2 ☐☐<br>জানিনা 997	

NO.	QUESTIONS AND FILTERS	CODING CATEGORIES			SKIP
653	এবার আমি আপনাকে _____এর চিকিৎসা বাবদ যত খরচ হয়েছিল সে সম্পর্কে বিস্তারিত জিজ্ঞাসা করবো । খরচ না হলে 0000 লিখুন।<br>(নাম)				
	মোট খরচের বিভাজন	১ম ভিজিট	২য় ভিজিট	৩য় ভিজিট	
	a. টিকিট বাবদ খরচ				
	b. ডায়াগনস্টিক টেস্ট (রক্ত, প্রস্রাব পরীক্ষা, আল্ট্রাসোনোগ্রাম ইত্যাদি) বাবদ খরচ				
	c. অপারেশন চার্জ বাবদ খরচ				
	d. ডাক্তারের ফি বাবদ খরচ				
	e. ঔষধ/ইনজেকশান বাবদ খরচ				
	f. হাসপাতাল বেড চার্জ/কেবিন চার্জ বাবদ খরচ				
	g. রক্ত, অক্সিজেন সিলিন্ডার বাবদ খরচ				
	h. বখশিস/দালাল বাবদ খরচ				
	i. _____এর খাবার বাবদ খরচ<br>(নাম)				
	j. অন্যান্য খরচ				
	k.এখানে আসার আগে বাড়ীতে চিকিৎসা বাবদ আর কোন খরচ				
653a	এবার আমি আপনাকে _____এর চিকিৎসার সময় আপনার সাথে যে ছিল তার খরচ সম্পর্কে বিস্তারিত জিজ্ঞাসা করবো।<br>(নাম)				
	মোট খরচের বিভাজন	১ম ভিজিট	২য় ভিজিট	৩য় ভিজিট	
	a. সঙ্গী/এটেন্ডেট এর থাকার ভাড়া বাবদ খরচ				
	b.সঙ্গী/এটেন্ডেট এর যাতায়াত বাবদ খরচ				
	c.সঙ্গী/এটেন্ডেট এর খাওয়া বাবদ খরচ				
	d.সঙ্গী/এটেন্ডেট বাবদ আর কোন খরচ				
	_____ এর **অসুস্থতার সময়ে আপনার কাজের যে ক্ষতি হয়েছে সে সর্ম্পকে কিছু প্রশ্ন করবো।**<br>(নাম)				
		১ম ভিজিট	২য় ভিজিট	৩য় ভিজিট	
654	_____ এর **অসুস্থতার সময়ে** আপনি মোট কত দিন<br>(নাম)<br>কাজে যেতে/করতে পারেন নাই?<br>**উত্তর না হলে 00 লিখে 655 প্রশ্নে যান।**	দিন.....	দিন.....	দিন.....	
654a	কাজে না যাওয়ার জন্যে ওই সময়ে আপনার আনুমানিক কত টাকা ক্ষতি হয়েছিল?<br>ক্ষতি **না হলে 0000 লিখুন।**	টাকা	টাকা	টাকা	
655	_____ **এর** অসুস্থতার সময়ে যিনি আপনার সাথে<br>(নাম)<br>ছিলেন, ওই সময়ে তিনি মোট কত দিন কাজে যেতে পারেন নাই? **উত্তর না হলে 00 লিখে 656 প্রশ্নে যান।**	দিন.....<br>সঙ্গী **ছিল** না.... 95<br>**(656 এ যান)**	দিন ....<br>সঙ্গী **ছিল** না.....95<br>**(656 এ যান)**	দিন.....<br>সঙ্গী **ছিল** না.....95<br>**(656 এ যান)**	
655a	এই কয় দিন কাজ না করার জন্যে তার মোট কত টাকা ক্ষতি হয়েছিল? ক্ষতি **না হলে 0000 লিখুন।**	টাকা	টাকা	টাকা	

NO.	QUESTIONS AND FILTERS	CODING CATEGORIES	SKIP
**656**	**সাক্ষাৎকারগ্রহনকারীঃ ২য়/৩য় বা তার অধিক ভিজিট করে থাকলে পুনরায় প্রশ্ন 647 থেকে জিজ্ঞেস করুন, অন্যথায় 657 প্রশ্নে যান।**		
657	_____এর চিকিৎসার জন্যে সব কিছু মিলিয়ে আপনার (নাম) সর্বমোট কত টাকা খরচ হয়েছিল?	টাকা.................... ☐☐☐☐☐☐	
658	_____এর চিকিৎসার জন্যে যে টাকা খরচ হয়েছিল, তা (নাম) কিভাবে যোগাড় করেছিলেন?	ঋণ/ধার করে ....A<br>সঞ্চয় থেকে বা বাড়ীর অন্যান্য খরচ বাঁচিয়ে.................B<br>সম্পত্তি/মূল্যবান জিনিষ বিক্রয় করে ....C<br>আত্মীয়/বন্ধু এর কাছ থেকে সাহায্য হিসাবে ....D<br>অন্যান্য _____________ ....X<br>(নির্দিষ্ট করুন)	B, C, D, X → **660**
658a	আপনি কোথা থেকে ধার করেছিলেন/ঋণ নিয়েছিলেন?	মাইক্রোক্রেডিট সংস্থা (এন জি ও) ....A<br>গ্রামের মহাজন...B<br>আত্মীয়..C<br>প্রতিবেশী...D<br>গ্রামের লোক ....E<br>অন্যান্য ________________________.............X<br>(নির্দিষ্ট করুন)	
658b	আপনারা যে টাকা ধার করেছিলেন তার জন্য কোন সুদ ধরা হয়েছিল কি?	হ্যাঁ ....1<br>না 2	2 → **660**
658c	আপনারা যে টাকা ধার করেছিলেন তার জন্য কত টাকা সুদ ধরা হয়েছে?<br>**(বলতে না পারলে জিজ্ঞাসা করুন যে পরিবারের কেউ জানে কিনা, কেউ জানলে তার কাছ থেকে শুনে লিখুন)।**<br>**হুবহুঃ** ______________________________<br>______________________________<br>______________________________<br>**(660 এ যান)**	☐☐	
659	কেন আপনি _____এর এই অসুস্থতার জন্য কোন (নাম) চিকিৎসা করান নি?<br>**জিজ্ঞেস করুনঃ আরও কিছু?**<br>**সব উত্তরের কোড বৃত্তায়িত করুন?**<br>**উত্তর একাধিক হতে পারে।**	অনেক দূরে ....A<br>সুবিধাজনক সময়ে সেবা দেওয়া হয় না ....B<br>সেবা প্রদানকারীর ব্যবহার ভাল নয়...........................C<br>সেবা প্রদানকারী দক্ষ নয়.......................................D<br>পর্যাপ্ত ওষুধপত্র পাওয়া যায় না ....E<br>অনেকক্ষণ অপেক্ষা করতে হয় ....F<br>ব্যয়বহুল..G<br>ধর্মীয় কারণ..H<br>যাওয়া দরকার এটা বুঝতে পারি নি............................I<br>পরিবারের অনুমতি ছিল না ....J<br>কোথায় স্বাস্থ্য সেবা দেয়া হয় জানতাম না..................K<br>অন্যান্য_______________ ....X<br>(নির্দিষ্ট করুন)	
660	______এর অসুস্থতার সময় মা-মনি স্বাস্থ্যকর্মী/কমিউনিটি (নাম) স্বাস্থ্যকর্মী (CHW) কোন ব্যবস্থা নিয়েছিলেন কি?	হ্যাঁ ....1<br>না 2	2 → **701**
660a	মা-মনি স্বাস্থ্যকর্মী/কমিউনিটি স্বাস্থ্যকর্মী (CHW) কি কি ব্যবস্থা নিয়েছিলেন?	নিয়মিত পরিদর্শন এবং স্বাস্থ্য পরীক্ষা করেছিলেন............A<br>হাসপাতালে ভর্তির পরামর্শ দিয়েছিলেন........................B<br>হাসপাতালে ভর্তির ব্যবস্থা করেছিলেন.........................C<br>অন্যান্য _______________...............................X<br>(নির্দিষ্ট করুন)	

Section G: Health Expenditure

সাক্ষাৎকারগ্রহণকারীঃ ০১ নভেম্বর ২০১১ থেকে ৩১ জানুয়ারী ২০১৩ এর মধ্যে উত্তরদাতার সর্বশেষ গর্ভের ডেলিভারির সময় এবং ডেলিভারির পরে মা'য়ের স্বাস্থ্য সেবা সংক্রান্ত খরচ সর্ম্পকে জিজ্ঞেস করতে হবে, সুতরাং উত্তরদাতাকে সেই গর্ভ সম্পর্কে ভাল করে বুঝিয়ে তারপর প্রশ্ন জিজ্ঞেস করুন।

Delivery Related Cost:

NO.	QUESTIONS AND FILTERS	CODING CATEGORIES	SKIP
**701**	**সাক্ষাৎকারগ্রহনকারীঃ প্রশ্ন 408 দেখুন এবং সঠিক কোড বৃত্তায়িত করুন।**	**কোড 11 বা 12 বা 13 বা 43 বা 96 বৃত্তায়িত........1**<br>**কোড 21 থেকে কোড 42 এর যে কোন একটি বৃত্তায়িত2**	 **→703a**
	**এখন আমি আপনার ডেলিভারির সময় স্বাস্থ্য সেবা সংক্রান্ড় খরচ সর্ম্পকে জানতে চাইব।**		
701a	____ এর জন্মের সময় ডেলিভারি বাবদ আপনাদের<br>(নাম)<br>মোট কত টাকা খরচ হয়েছিল?	টাকা............................. ☐☐☐☐☐	
701b	_____ এর জন্মের সময় ডেলিভারি বাবদ যে খরচ<br>(নাম)<br>হয়েছে সে সম্পর্কে এখন আমি আলাদা আলাদা ভাবে জানতে চাই, ____ কত টাকা খরচ হয়েছে ?<br>(বিষয়)<br>**(প্রত্যেকটি জিজ্ঞেস করুন)।**<br>**(কোন খরচ না হলে 0000 লিখুন; বলতে না পারলে জিজ্ঞাসা করুন যে পরিবারের কেউ জানে কিনা, জানলে তার কাছ থেকে শুনে লিখুন, না হলে 9997 লিখুন)।**	বিভাজন — মোট খরচ (টাকা)<br>a. সেবাপ্রদানকারী বাবদ ☐☐☐☐<br>b. ঔষধ বাবদ ☐☐☐☐<br>c. ডেলিভারির চিকিৎসা সংক্রান্ড় অন্যান্য খরচ বাবদ ☐☐☐☐<br>d. অন্যান্য খরচ (বখশিস,মিষ্টি ইত্যাদি) বাবদ ☐☐☐☐	
**702**	**সাক্ষাৎকারগ্রহনকারীঃ প্রশ্ন 406 দেখুন এবং সঠিক কোড বৃত্তায়িত করুন।**	**এক বা একাধিক কোড 1 বৃত্তায়িত ....1**<br>**সবগুলো কোড 2 বৃত্তায়িত 2**	 **→717**
703	আপনি বলেছেন ডেলিভারির সময় আপনার __________ সমস্যা<br>(406 এর উত্তর)<br>হয়েছিল। এর জন্য আপনি কোন চিকিৎসা করিয়েছেন কি?	হ্যাঁ .... 1<br>না.. 2<br>জানি না/মনে নেই.. 7	**→704a**<br>**→717**
**703a**	**সাক্ষাৎকারগ্রহনকারীঃ প্রশ্ন 406 দেখুন এবং সঠিক কোড বৃত্তায়িত করুন।**	**এক বা একাধিক কোড 1 বৃত্তায়িত ....1**<br>**সবগুলো কোড 2 বৃত্তায়িত 2**	 **→ 704b**
704	আপনি বলেছেন ডেলিভারির সময় আপনার__________<br>(406 এর উত্তর)<br>সমস্যা হয়েছিল। এর জন্য আপনি কোন চিকিৎসা করিয়েছেন কি?	হ্যাঁ .... 1<br>না.. 2<br>জানি না/মনে নেই.. 7	 **→704b**
704**a.**	আপনি কার কার কাছ থেকে চিকিৎসা নিয়েছেন?<br><br>**কোন উত্তর বলবেন না।**<br><br>**জিজ্ঞেস করুনঃ আরও কিছু?**<br><br>**সব উত্তরের কোড বৃত্তায়িত করুন।**	পাশ করা (MBBS) ডাক্তার .... A<br>নার্স/ধাত্রী.. B<br>প্যারামেডিক .... C<br>পরিবার কল্যাণ পরিদর্শিকা (FWV) .... D<br>কম্যুউনিটি স্কিল্ড বার্থ এটেন্টডেন্ট (CSBA)............................ E<br>উপসহকারী কমিউনিটি চিকিৎসা কর্মকর্তা ....F<br>মা-মনি স্বাস্থ্যকর্মী/ CHW .... G<br>স্বাস্থ্য সহকারী (HA)... H<br>পরিবার কল্যাণ সহকারী (FWA) .... I<br>কমিউনিটি হেলথ কেয়ার প্রোভাইডার (CHCP)................. J<br>প্রশিক্ষণপ্রাপ্ত টিবিএ (TTBA) ....K<br>প্রশিক্ষণহীন টিবিএ (ধন্নী, চাউনি, দাই) ....L<br>হোমিওপ্যাথ.. M<br>আয়ুর্বেদিক চিকিৎসক .... N<br>হাতুরে ডাক্তার/কোয়াক.. O<br>গ্রাম ডাক্তার/পল-ী চিকিৎসক..P<br>ওঝা/ কবিরাজ... Q<br>অন্যান্য স্বাস্থ্যকর্মী .... R<br>অন্যান্য__________ ....X<br>(নির্দিষ্ট করুন)<br>জানি না/মনে নাই ....Z	

NO.	QUESTIONS AND FILTERS	CODING CATEGORIES	SKIP
704b.	ডেলিভারির জন্য (এবং __________ এর চিকিৎসার জন্য)<br>(406 এর উত্তর)<br>আপনাকে মোট কতবার (ভর্তি ছাড়া এবং ভর্তি সহ) ডাক্তারের কাছে/স্বাস্থ্যকেন্দ্রে/হাসপাতালে যেতে হয়েছিল?	বার .... [ ]<br>স্বাস্থ্যকেন্দ্রে যাইনি, বাড়ীতেই চিকিৎসা নিয়েছি 9	→ **716a**
705	ডেলিভারির (এবং চিকিৎসার) জন্য আপনাকে স্বাস্থ্যকেন্দ্রে/ হাসপাতালে ভর্তি হতে হয়েছিল কি?	হ্যাঁ .... 1<br>না 2	→ **706**
705a	আপনাকে কোন্ স্বাস্থ্যকেন্দ্রে/ হাসপাতালে ভর্তি হতে হয়েছিল?	**সরকারী স্বাস্থ্যকেন্দ্র**<br>মেডিকেল কলেজ হাসপাতাল.................................A<br>জেলা/সদর হাসপাতাল..B<br>মা ও শিশু স্বাস্থ্যকেন্দ্র..C<br>উপজেলা স্বাস্থ্য কমপে- ক্স ....D<br>ইউনিয়ন স্বাস্থ্য ও পরিবার কল্যাণ কেন্দ্র/<br>সাব সেন্টার/আরডি.. E<br>কমিউনিটি ক্লিনিক..F<br>**এনজিও স্বাস্থ্যকেন্দ্র**<br>এনজিও হাসপাতাল ....G<br>এনজিও স্থায়ী স্বাস্থ্যকেন্দ্র ....H<br>বেসরকারী/ প্রাইভেট হাসপাতাল/ ক্লিনিক...................... I<br>অন্যান্য প্রাইভেট স্বাস্থ্যকেন্দ্র .... J<br>অন্যান্য _______________ ....X<br>(নির্দিষ্ট করুন)	
705b	আপনাকে স্বাস্থ্যকেন্দ্রে/হাসপাতালে মোট কতবার ভর্তি হতে হয়েছিল?	বার .... [ ]	
705c	আপনি (প্রতিবার) কতদিন ভর্তি ছিলেন?	দিন<br>প্রথমবার... [ ][ ]<br>দ্বিতীয়বার .... [ ][ ]<br>তৃতীয়বার .... [ ][ ]	

NO.	QUESTIONS AND FILTERS	CODING CATEGORIES			SKIP
**706**	**সাক্ষাৎকারগ্রহনকারীঃ প্রশ্ন 704b দেখুন। উত্তর একবার হলে শুধু মাত্র ১ম ভিজিটের কলাম, দুইবার হলে ১ম ও ২য় ভিজিটের কলাম, তিনবার হলে তিনটি কলামই 707 থেকে 714a পর্যন্ড় প্রশ্নগুলো জিজ্ঞেস কর‍ুন। তিনবারের অধিক হলে অতিরিক্ত শীট ব্যবহার কর‍ুন।**				
	**আপনার ডেলিভারির (ও ডেলিভারি সংক্রান্ড় জটিলতার চিকিৎসার) জন্য আপনি মোট____ বার ডাক্তারের কাছে/স্বাস্থ্যকেন্দ্রে/ হাসপাতালে গিয়েছিলেন। আমি এখন আপনার কাছ থেকে (প্রত্যেকবারের) খরচ আলাদা আলাদা করে জানতে চাই। (সাক্ষাৎকারগ্রহনকারীঃ উত্তরদাতা বলতে না পারলে পরিবারের অন্যান্যদের সাহায্য নিন।)**				
		১ম ভিজিট	২য় ভিজিট	৩য় ভিজিট	
707	ডেলিভারির (ও ডেলিভারি সংক্রান্ড় জটিলতার) জন্য যে ডাক্তারের কাছে/স্বাস্থ্যকেন্দ্রে/হাসপাতালে গিয়েছিলেন, আপনার বাড়ী থেকে সেটির দূরত্ব কত?	☐☐ কি মি	☐☐ কি মি	☐☐ কি মি	
708	ওই ডাক্তারের কাছে/স্বাস্থ্যকেন্দ্রে/হাসপাতালে আপনি কিসে গিয়েছিলেন?<br>**(যদি একাধিক যানবাহন ব্যবহার করে থাকেন তাহলে সব থেকে বেশী দূরত্ব অতিক্রম করতে যে যানবাহন ব্যবহার করেছেন সে সম্পর্কে জিজ্ঞেস কর‍ুন।)**	রিকশা/ ভ্যান.......01<br>বাস ....02<br>টেম্পু ....03<br>মটরসাইকেল ......04<br>সি এন জি ....05<br>এম্বুলেন্স ....06<br>নৌকা ....07<br>পায়ে হেঁটে ....08<br>অন্যান্য ______..96<br>(নির্দিষ্ট করুন)	রিকশা/ ভ্যান ...... 01<br>বাস .... 02<br>টেম্পু .... 03<br>মটরসাইকেল ..... 04<br>সি এন জি .... 05<br>এম্বুলেন্স .... 06<br>নৌকা .... 07<br>পায়ে হেঁটে .... 08<br>অন্যান্য ______.. 96<br>(নির্দিষ্ট করুন)	রিকশা/ ভ্যান.......01<br>বাস ....02<br>টেম্পু ....03<br>মটরসাইকেল ......04<br>সি এন জি ....05<br>এম্বুলেন্স ....06<br>নৌকা ....07<br>পায়ে হেঁটে ....08<br>অন্যান্য ______...96<br>(নির্দিষ্ট করুন)	
708a	আপনার বাড়ী থেকে ওই ডাক্তারের কাছে/স্বাস্থ্যকেন্দ্রে/ হাসপাতালে যেতে কত সময় লেগেছিল? (যাতায়াতের মোট সময় এবং যানবাহনের জন্য অপেক্ষার সময় সহ)<br>**১ ঘন্টার কম হলে মিনিটে লিখুন।**<br>**১ ঘন্টার বেশি হলে পূর্ণ ঘন্টায় লিখুন।**	মিনিট. 1 ☐☐<br>**ঘন্টা ...**2 ☐☐<br>জানিনা......... 997	মিনিট .1 ☐☐<br>**ঘন্টা ...**2 ☐☐<br>জানিনা 997	মিনিট . 1 ☐☐<br>**ঘন্টা ..** 2 ☐☐<br>জানিনা 997	
709	ওই ডাক্তারের কাছে/স্বাস্থ্যকেন্দ্রে/হাসপাতালে যেতে আপনার মোট কত টাকা খরচ হয়েছিল?<br>(কোন খরচ না হলে 0000 লিখুন; বলতে না পারলে জিজ্ঞাসা কর‍ুন যে পরিবারের কেউ জানে কিনা, জানলে তার কাছ থেকে শুনে লিখুন, না হলে 9997 লিখুন)।	টাকা ☐☐☐☐	টাকা ☐☐☐☐	টাকা ☐☐☐☐	
710	ওই ডাক্তারের কাছ/স্বাস্থ্যকেন্দ্র/হাসপাতাল থেকে ফেরার সময় আপনার মোট কত টাকা খরচ হয়েছিল?<br>(কোন খরচ না হলে 0000 লিখুন; বলতে না পারলে জিজ্ঞাসা কর‍ুন যে পরিবারের কেউ জানে কিনা, জানলে তার কাছ থেকে শুনে লিখুন, না হলে 9997 লিখুন)।	টাকা ☐☐☐☐	টাকা ☐☐☐☐	টাকা ☐☐☐☐	
711	ডাক্তারের কাছে/স্বাস্থ্যকেন্দ্রে/হাসপাতালে পৌঁছাবার পর থেকে চিকিৎসা পাবার আগ পর্যন্ড় আপনাকে মোট কত সময় অপেক্ষা করতে হয়েছিল?	মিনিট. 1 ☐☐<br>ঘন্টা . 2 ☐☐<br>জানিনা......... 997	মিনিট .1 ☐☐<br>ঘন্টা ..2 ☐☐<br>জানিনা 997	মিনিট . 1 ☐☐<br>ঘন্টা .. 2 ☐☐<br>জানিনা 997	

712	এবার আমি আপনাকে ডেলিভারির (ও ডেলিভারি সংক্রান্ড় জটিলতার) জন্য যত খরচ হয়েছিল সে সম্পর্কে বিস্ড়রিত জিজ্ঞাসা করবো । **কোন খরচ না হলে 0000 লিখুন।**				
	মোট খরচের বিভাজন	১ম ভিজিট	২য় ভিজিট	৩য় ভিজিট	
	a. টিকিট বাবদ খরচ				
	b. ডায়াগনস্টিক টেস্ট (রক্ত, প্রস্রাব পরীক্ষা, আল্ট্রাসোনোগ্রাম ইত্যাদি) বাবদ খরচ				
	c. অপারেশন চার্জ বাবদ খরচ				
	d. ডাক্তারের ফি বাবদ খরচ				
	e. ঔষধ/ইনজেকশান বাবদ খরচ				
	f. হাসপাতাল বেড চার্জ/কেবিন চার্জ বাবদ খরচ				
	g. রক্ত, অক্সিজেন সিলিন্ডার বাবদ খরচ				
	h. বখশিস / দালাল বাবদ খরচ				
	i. আপনার খাবার বাবদ খরচ				
	j. অন্যান্য খরচ				
	k.এখানে আসার আগে বাড়ীতে চিকিৎসা বাবদ আর কোন খরচ				
712a	আপনার সাথে যে ছিল তার খরচ সম্পর্কে এখন আমি বিস্ড়রিত জিজ্ঞাসা করবো । **কোন খরচ না হলে 0000 লিখুন।**				
	মোট খরচের বিভাজন	১ম ভিজিট	২য় ভিজিট	৩য় ভিজিট	
	a. সঙ্গী/ এটেন্ডেট এর থাকার ভাড়া বাবদ খরচ				
	b. সঙ্গী/ এটেন্ডেট এর যাতায়াত বাবদ খরচ				
	c. সঙ্গী/ এটেন্ডেট এর খাওয়া বাবদ খরচ				
	d. সঙ্গী/ এটেন্ডেট বাবদ আর কোন খরচ				
	**ডেলিভারির (এবং জটিলতার) জন্যে আপনার এবং আপনার সঙ্গীর কাজের যে ক্ষতি হয়েছে সে সর্ম্পকে কিছু প্রশ্ন জিজ্ঞাসা করবো।**				
		১ম ভিজিট	২য় ভিজিট	৩য় ভিজিট	
713	আপনি ডেলিভারির (এবং জটিলতার) জন্যে মোট কত দিন কাজে যেতে পারেন নাই?<br>**উত্তর না হলে 000 লিখে 714 প্রশ্নে যান।**	দিন …	দিন …	দিন …	
713a	**এই কাজে না যাওয়ার জন্যে ওই সময়ে আনুমানিক কত টাকা ক্ষতি হয়েছিল?**	টাকা	টাকা.	টাকা.	
714	**ডেলিভারির (এবং জটিলতার)** সময় যিনি **আপনার সাথে ছিলেন, ওই সময়ে তিনি** মোট কত দিন কাজে যেতে পারে নাই?<br>**উত্তর না হলে 00 লিখে 715** প্রশ্নে যান।	দিন ………	দিন …..	দিন …..	
714a	এই কয় দিন কাজ না করার জন্যে তার মোট কত টাকা ক্ষতি হয়েছিল?<br>**উত্তর না হলে 0000 লিখুন।**	টাকা	টাকা	টাকা	
**715**	**সাক্ষাৎকারগ্রহনকারীঃ ২য়/৩য় বা তার অধিক ভিজিট করে থাকলে পুনরায় প্রশ্ন 707 থেকে জিজ্ঞেস কর্ন, অন্যথায় 716 প্রশ্নে যান।**				

NO.	QUESTIONS AND FILTERS	CODING CATEGORIES	SKIP
716	আপনার ডেলিভারির (ও ডেলিভারি সংক্রান্ড় জটিলতার) জন্য সব কিছু মিলিয়ে সর্বমোট কত টাকা খরচ হয়েছিল?	টাকা..........................☐☐☐☐☐☐	→ 717
716a	________এর চিকিৎসার জন্য আপনার মোট কত (406 এর উত্তর) টাকা খরচ হয়েছিল?	টাকা ....☐☐☐☐☐	
717	আপনার ডেলিভারির (এবং ডেলিভারি সংক্রান্ড় জটিলতার) জন্যে যে টাকা খরচ হয়েছিল, তা কিভাবে যোগাড় করেছিলেন?	ঋণ/ধার করে ....A<br>সঞ্চয় থেকে বা বাড়ীর অন্যান্য খরচ বাঁচিয়ে......................B<br>সম্পত্তি/মূল্যবান জিনিষ বিক্রয় করে .... C<br>আত্মীয়/বন্ধু এর কাছ থেকে সাহায্য হিসাবে ....D<br>অন্যান্য ______________ ....X<br>(নির্দিষ্ট করুন)	B–X → 800
717a	আপনি কোথা থেকে ধার করেছিলেন/ঋণ নিয়েছিলেন?	মাইক্রোক্রেডিট সংস্থা (এন জি ও)................................ A<br>গ্রামের মহাজন.. B<br>আত্মীয় .... C<br>প্রতিবেশী... D<br>গ্রামের লোক ....E<br>অন্যান্য _______________________ .... X<br>(নির্দিষ্ট করুন)	
717b	আপনারা যে টাকা ধার করেছিলেন তার জন্য কোন সুদ ধরা হয়েছিল কি?	হ্যাঁ .... 1<br>না 2	2 → 800
717c	আপনারা যে টাকা ধার করেছিলেন তার জন্য কত টাকা সুদ ধরা হয়েছে?<br>**(বলতে না পারলে জিজ্ঞাসা করুন যে পরিবারের কেউ জানে কিনা, কেউ জানলে তার কাছ থেকে শুনে লিখুন)।**<br>**হুবহুঃ** ______________________________<br>______________________________<br>______________________________	☐☐	

Section H: Previous Birth Related Questions

এতক্ষণ আপনার যে বাচ্চা/গর্ভ সম্বন্ধে কথা বলছিলাম, সেই বাচ্চার/গর্ভের আগের গর্ভ সম্পর্কে এখন আমি আপনার সাথে কথা বলতে চাই। অর্থাৎ ০১ **নভেম্বর** ২০১১ থেকে ৩১ জানুয়ারী ২০১৩ এর মধ্যে হওয়া সর্বশেষ গর্ভের আগের গর্ভ সম্পর্কে চিন্তা করুন। এখন আমি সেই গর্ভ সম্বন্ধে জিজ্ঞেস করব।

সাক্ষাৎকারগ্রহনকারীঃ ০১ **নভেম্বর** ২০১১ থেকে ৩১ জানুয়ারী ২০১৩ এর মধ্যে হওয়া সর্বশেষ গর্ভের আগের গর্ভের সন্তানের নাম জিজ্ঞেস করুন এবং নাম ধরে প্রশ্ন করুন। যদি যমজ বা ২ এর অধিক সন্তান সর্বশেষ গর্ভের আগের গর্ভে হয়, তাহলে আরেকটি প্রশ্নপত্র নিয়ে বাকি সন্তান এর সম্পর্কিত তথ্য Section H এ লিখুন এবং এই প্রশ্নপত্রের সাথে যুক্ত করুন।

NO.	QUESTIONS AND FILTERS	CODING CATEGORIES	SKIP
800	____________এর আগে আপনার যে বাচ্চা জন্মেছে<br>(সর্বশেষ বাচ্চার নাম)<br>তার নাম কি?	নামঃ ______________________<br>**আর কোন বাচ্চা জন্মায় নি..............................6**	**→901**
801	____এর জন্ম কবে হয়েছিল?<br>(নাম)	☐☐ ☐☐ ☐☐☐☐<br>**দিন মাস সাল**	
802	____এর জন্ম কি সিজারিয়ান অপারেশনের মাধ্যমে<br>(নাম)<br>হয়েছিল?	হ্যাঁ .... 1<br>না 2	
803	____এর জন্মের সময় আপনার প্রসব ব্যথা ১২ ঘন্টার<br>(নাম)<br>বেশী ছিল কি?	হ্যাঁ .... 1<br>না .... 2<br>জানি না 7	
804	____ পেটে থাকাকালীন সময়ে আপনার কোন সমস্যা/অসুবিধা/জটিলতা হয়েছিল কি যার জন্য চিকিৎসার প্রয়োজন ছিল?	হ্যাঁ .... 1<br>না .... 2<br>মনে নাই 7	2, 7 **→806**
805	আপনার কি ধরণের সমস্যা/অসুবিধা/জটিলতা হয়েছিল?<br><br>**একাধিক উত্তর হতে পারে।**<br><br>**সব উত্তরের কোড বৃত্তায়িত করুন।**	তীব্র মাথা ব্যথা .... A<br>চোখে ঝাপসা দেখা .... B<br>যোনী পথে অতিরিক্ত রক্তস্রাব .... C<br>জ্বর.. D<br>খিঁচুনী/ফিট .... E<br>হাতে পানি আসা/ ফুলে যাওয়া.. F<br>মুখমন্ডলে পানি আসা/ফুলে যাওয়া.. G<br>গর্ভের বাচ্চার নড়াচড়া কমে যাওয়া/বন্ধ হওয়া .... H<br>তলপেটে তীব্র ব্যথা .... I<br>পায়ে পানি আসা .... J<br>উচ্চ রক্তচাপ.. K<br>সময় পূর্ণ হওয়ার আগে পানি ভাঙ্গা .... L<br>অচেতন হওয়া/জ্ঞান হারিয়ে ফেলা .... M<br>কষ্ট করে শ্বাস নেয়া .... N<br>প্রচন্ড দুর্বলতা .... O<br>অতিরিক্ত বমি .... P<br>অতিরিক্ত সাদা স্রাব .... Q<br>অন্যান্য__________ .... X<br>(নির্দিষ্ট করুন)<br>মনে নাই/কিছুই উলে- খ করেন নি .... Y	
806	_____এর জন্মের সময় আপনার কোন<br>(নাম)<br>সমস্যা/অসুবিধা/জটিলতা হয়েছিল কি?	হ্যাঁ .... 1<br>না 2	2 **→ 808**

NO.	QUESTIONS AND FILTERS	CODING CATEGORIES	SKIP
807	_____এর জন্মের সময় আপনার কি ধরণের<br>(নাম)<br>সমস্যা/অসুবিধা/জটিলতা হয়েছিল?<br><br>**সব উত্তরের কোড বৃত্তায়িত কর ুন।**<br><br>**উত্তর একাধিক হতে পারে।**	বাচ্চা হওয়ার রাস্তা (যোনী পথে)<br>দিয়ে অতিরিক্ত রক্ত গিয়েছিল..........................A<br>দুর্গন্ধযুক্ত স্রাব গিয়েছিল ....B<br>তীব্র জ্বর হয়েছিল ....C<br>শিশুর হাত পা আগে বের হয়ে এসেছিল..............D<br>(পেটের মধ্যে) শিশুর অস্বাভাবিক অবস্থান ছিল....... E<br>দীর্ঘ প্রসব (১২ ঘন্টার বেশি) ব্যথা ছিল................ F<br>প- াসেন্টা বা ফুল পড়ে নি..............................G<br>ইউটেরাস বা গর্ভদানী/জন্ম দ্বার ছিঁড়ে গিয়েছিল ....H<br>(শিশুর) নাড়ী বেরিয়ে এসেছিল .... I<br>(শিশুর গলায়) নাড়ী পেঁচিয়ে গিয়েছিল..................J<br>খিঁচুনী হয়েছিল ....K<br>তীব্র মাথা ব্যথা হয়েছিল .... L<br>যোনী পথ দিয়ে সবুজাভ কিছু বের হয়েছিল .... M<br>পা/মুখ ফুলে গিয়েছিল...................................N<br>সময়ের আগে পানি ভেঙ্গেছিল...........................O<br>অন্যান্য ____________.............................. .X<br>(নির্দিষ্ট কর ুন)	
808	______ জন্মের পর তার ওজন নেয়া হয়েছিল কি?<br>(নাম)	হ্যাঁ ....1<br>না ....2<br>মনে নাই 7	2, 7 → **810**
809	ওজন কত হয়েছিল?<br><br>**কার্ড দেখাতে পারলে, কার্ড থেকে ওজন লিখুন।**<br>**কার্ড না দেখাতে পারলে, শুনে ওজন লিখুন।**	জন্ম ওজন<br>কেজি ....1 [ ].[ ][ ]<br>পাউন্ড ....2 [ ].[ ][ ]<br>জানি না/মনে নাই..................................9997	
810	জন্মের পর _____ আকারে কতটুকু ছিল?<br>(নাম)<br>**স্বাভাবিকের চেয়ে অনেক ছোট, নাকি স্বাভাবিকের চেয়ে একটু ছোট, নাকি স্বাভাবিক, নাকি স্বাভাবিকের চেয়ে বড়?**	অনেক ছোট..1<br>স্বাভাবিকের থেকে ছোট ....2<br>স্বাভাবিক ....3<br>স্বাভাবিকের থেকে বড়.....................................4<br>ছোট না বড়, বুঝি নাই................................ 7	
811	______কে কি হামের টিকা দেয়া হয়েছিল, যা ৯ মাস<br>(নাম)<br>শেষে ১০ মাস বয়সে বাচ্চার রানের মাংসে দেয়া হয়?	হ্যাঁ ....1<br>না ....2<br>জানি না 7	

Section I: Knowledge

এখন আমি আপনার কাছে নবজাতকের যত্ন সম্পর্কে জানতে চাইব।

NO.	QUESTIONS AND FILTERS	CODING CATEGORIES	SKIP
901	জন্মের ১ মাসের মধ্যে বাচ্চার কি কি স্বাস্থ্য সমস্যা/অসুবিধা হতে পারে যার জন্য ডাক্তারী চিকিৎসার প্রয়োজন হয়?<br><br>**জিজ্ঞেস করুনঃ আরও কিছু?**<br><br>**উত্তর পড়ে শোনাবেন না।**<br><br>**সব উত্তরের কোড বৃত্তায়িত করুন।**	কষ্টকর/দ্রুত শ্বাস নেয়া ....A<br>নিউমোনিয়া ....B<br>ঠান্ডা/কফ/সর্দি/কাশি ....C<br>চামড়ার রং, হাত, হাতের তালু, পায়ের পাতা, চোখ হলুদ হওয়া/জন্ডিস/ওলমি ....D<br>বাচ্চার খাওয়া কমে যাওয়া/বুকের দুধ চুষতে না পারা ....E<br>নাভির চারপাশে লাল হওয়া/কিছু বের হওয়া ....F<br>চামড়ায় ফোসকা/ঘা হওয়া ....G<br>খিঁচুনী/শরীর শক্ত ....H<br>অচেতন/অজ্ঞান/হুঁশ না থাকা ....I<br>চোখ লাল হওয়া/ময়লা বা পিঙ্গিস বের হওয়া ....J<br>বাচ্চার শরীর ঠান্ডা হওয়া ....K<br>বাচ্চা না কাঁদা ....L<br>জ্বর ....M<br>প্রস্রাব না হওয়া ....N<br>পায়খানা না করা ....O<br>একটানা বমি ....P<br>পেট ফাঁপা ....Q<br>ঘুম থেকে জাগানো কষ্টকর ....R<br>চামড়ায় ফুসকুড়ি/র‍্যাশ/মাসিপিসি ....S<br>হাম / প্যারা / ফ্যারা ....T<br>ডায়রিয়া ....U<br>বুকের খাঁচা ডেবে যাওয়া বা ভিতরে ঢুকে যাওয়া ....V<br>অন্যান্য ________ ....X<br>**(নির্দিষ্ট করুন)**<br>জানি না ....Y	
902	আপনি কি কখনো মোবাইল ফোন ব্যবহার করেছেন?	হ্যাঁ ....1<br>না 2	2 → **1001**
903	আপনি কার মোবাইল ফোন ব্যবহার করেন?<br><br>**প্রোব করুন।**<br>**একাধিক উত্তর হতে পারে।**<br>**সব উত্তরের কোড বৃত্তায়িত করুন।**	নিজের ....A<br>স্বামী ....B<br>পরিবারের অন্য কেউ ....C<br>প্রতিবেশী ....D<br>দোকান ....E<br>অন্যান্য ________ ....X<br>(নির্দিষ্ট করুন)	B–X → **906**
904	আপনি কি সাধারনত সব সময় মোবাইল ফোন **চালু রাখেন?**	হ্যাঁ ....1<br>না 2	1 → **906**
905	দিনে কতক্ষণ আপনার মোবাইল ফোন বন্ধ রাখেন?	ঘন্টা ....[ ][ ]<br>অন্যান্য ________ ....96<br>(নির্দিষ্ট করুন)	
906	আপনি কি মোবাইল ফোনের মাধ্যমে কাউকে **ম্যাসেজ/ SMS** পাঠাতে পারেন বা কেউ **ম্যাসেজ/ SMS** পাঠালে পড়তে পারেন?	**ম্যাসেজ/ SMS** পাঠাতে পারি ....A<br>**ম্যাসেজ/ SMS** পড়তে পারি ....B<br>**ম্যাসেজ/ SMS** পাঠাতে পারি না ....C<br>**ম্যাসেজ/ SMS** পড়তে পারি না ....D	

NO.	QUESTIONS AND FILTERS	CODING CATEGORIES	SKIP
907	আপনার গর্ভকালীন সময়ে, _____এর জন্মের সময় বা<br>(নাম)<br>_____এর অসুস্থতার সময় কখনও কি মোবাইল ফোন<br>(নাম)<br>ব্যবহার করতে হয়েছিল?	হ্যাঁ... 1<br>না 2	2 → **1001**
907a	কোন্ সময়ে মোবাইল ফোন ব্যবহার করতে হয়েছিল?<br>**প্রোব করুন।**<br>**একাধিক উত্তর হতে পারে।**<br>**সব উত্তরের কোড বৃত্তায়িত করুন।**	গর্ভকালীন সময়ে...A<br>ডেলিভারীর সময় ....B<br>বাচ্চার অসুস্থতার সময় ....C<br>অন্যান্য _______________.................................X<br>(নির্দিষ্ট করুন)	
907b	___________কেন মোবাইল ফোন ব্যবহার করতে<br>(907a এর উত্তর)<br>হয়েছিল?	স্বামীকে খবর দেওয়ার জন্য.....................................A<br>জানতাম না কোথায়/ কার কাছে যেতে হবে................. B<br>টাকা পয়সা জোগাড় করার জন্য.............................. C<br>সরাসরি স্বাস্থ্যসেবাদানকারীর সাথে যোগাযোগের জন্য .... D<br>যানবাহনের সমস্যা... E<br>সাথে যাবার মত কেউ ছিল না .... F<br>স্বাস্থ্যকেন্দ্রে যাবার মত সময় ছিল না......................... G<br>এই অবস্থায় কি করণীয় জানার জন্য...........................H<br>স্বাস্থ্যকেন্দ্র বাসা হতে অনেক দুরে.............................. I<br>নিকট আত্মীয়কে খবর দেয়ার জন্য..............................J<br>অন্যান্য ________________...............................X<br>**(নির্দিষ্ট করুন)**	

Section J: Household Section

এখন আমি আপনার এবং আপনার খানার সম্পর্কে কিছু তথ্য জানতে চাই।

NO.	QUESTIONS AND FILTERS	CODING CATEGORIES	SKIP
1001	আপনাদের খানায় কোন্ বয়সের কতজন পুরুষ এবং মহিলা আছে বলুন?<br><br>**কোন বয়সের পুরুষ এবং মহিলা সদস্য না থাকলে বক্সে '00' লিখুন।**<br><br>**(পুরুষ এবং মহিলার সংখ্যা যোগ করে মোট এর বক্সে লিখুন।)**	**পুরুষ** **মহিলা**<br>0-4 বৎসর .... ☐☐ ☐☐<br>5-14 বৎসর .... ☐☐ ☐☐<br>15-29 বৎসর .... ☐☐ ☐☐<br>30-49 বৎসর .... ☐☐ ☐☐<br>> 50 বৎসর .... ☐☐ ☐☐<br>**মোট** .... ☐☐ ☐☐	
1002	থালা বাসন ধোয়ার জন্য প্রধানতঃ আপনারা কোথাকার পানি ব্যবহার করেন?	**পাইপের পানি ঃ**<br>বাড়ির ভিতরে ট্যাপের (পাইপের) পানি .... 11<br>বাড়ির বাহিরে ট্যাপের (পাইপের) পানি .... 12<br>**কূপের পানি ঃ**<br>নলকুপ .... 21<br>শ্যালো টিউবওয়েল .... 22<br>গভীর নলকূপ .... 23<br>কূয়া .... 24<br>**ভূ-পৃষ্ঠের পানি ঃ**<br>পুকুর/বদ্ধ জলাশয়/হ্রদ .... 31<br>নদী/খাল/ঝর্ণার পানি .... 32<br>বৃষ্টির পানি.. 41<br>অন্যান্য ______________________________ 96<br>(নির্দিষ্ট করুন)	
1003	আপনাদের খানায় কি ধরণের পায়খানা/ল্যাট্রিন এর ব্যবস্থা আছে?	সেপটিক ট্যাংক/আধুনিক ল্যাট্রিন .... 11<br>**গর্ত (পিট) টয়লেট/ল্যাট্রিনঃ**<br>জলাবদ্ধ/স্লাব (স্যানিটারী) ল্যাট্রিন .... 21<br>গর্তের (পিট) ল্যাট্রিন .... 22<br>খোলা/ঝুলন্ড় ল্যাট্রিন .... 23<br>ল্যাট্রিন নাই/ঝোপ-ঝাড়/মাঠ .... 31<br>অন্যান্য ______________________________ 96<br>(নির্দিষ্ট করুন)	
1004	আপনার খানায় বিদ্যুৎ আছে কি?	হ্যাঁ .... 1<br>না 2	

NO.	QUESTIONS AND FILTERS	CODING CATEGORIES				SKIP
1005	আপনার খানায় (বা খানার কোন সদস্যের) ______ (জিনিস) আছে কি?<br><br>প্রত্যেকটি জিনিস সম্বন্ধে আলাদা ভাবে জিজ্ঞেস করুন ।		জিনিস	হ্যাঁ	না	
		A.	আলমারী/ওয়ার্ডরোব	1	2	
		B.	টেবিল	1	2	
		C.	চেয়ার/বেঞ্চ	1	2	
		D.	চালু ঘড়ি/দেয়াল ঘড়ি	1	2	
		E.	খাট/চৌকি	1	2	
		F.	চালু রেডিও	1	2	
		G.	চালু টেলিভিশন	1	2	
		H.	ক্যাসেট পে- য়ার	1	2	
		I.	টেবিল ফ্যান/সিলিং ফ্যান	1	2	
		J.	লেপ/কম্বল	1	2	
		K.	তোষক/জাজিম	1	2	
		L.	চালু ফ্রিজ	1	2	
		M	সাইকেল	1	2	
		N.	মোটর সাইকেল	1	2	
		O.	চালু সেলাই মেশিন	1	2	
		P.	চালু টেলিফোন	1	2	
		Q.	চালু মোবাইল ফোন	1	2	
		R.	কার/মাইক্রোবাস/টেম্পু	1	2	
		S.	রিক্সা/রিক্সা-ভ্যান	1	2	
		T.	নৌকা	1	2	
1006	আপনাদের গৃহপালিত পশু আছে কি?	হ্যাঁ...1<br>না...2 →				1007
1006a	কয়টি _______আছে? (পশুপাখি)<br><br>**প্রত্যেকটি পড়ে শোনান।**<br><br>**জানিনা হলে 97 লিখুন।**<br><br>**না থাকলে 00 লিখুন।**		**পশুপাখি**	**সংখ্যা**		
		A.	গরু	[ ][ ]		
		B.	মহিষ	[ ][ ]		
		C.	ছাগল	[ ][ ]		
		D.	ভেড়া	[ ][ ]		
		E.	মুরগী	[ ][ ]		
		F.	হাঁস	[ ][ ]		

NO.	QUESTIONS AND FILTERS	CODING CATEGORIES	SKIP
1007	বসত ঘরের চালের/ছাদের প্রধান নির্মাণ-সামগ্রীঃ<br><br>**দেখে লিপিবদ্ধ কর ুন**	**কাঁচা ছাদ ঃ**<br>কাঁচা (বাঁশ/খড়) .... 11<br>**প্রাথমিক পর্যায়ের ছাদ ঃ**<br>টিন .... 21<br>**পাকা ছাদ ঃ**<br>সিমেন্ট/ইট বালি জমানো/টালি .... 31<br>অন্যান্য ____________.................................. 96<br>(নির্দিষ্ট কর ুন)	
1008	বসত ঘরের দেয়ালের প্রধান নির্মাণ-সামগ্রীঃ<br><br>**দেখে লিপিবদ্ধ কর ুন**	**কাঁচা দেয়াল ঃ**<br>পাটকাঠি/বাঁশ/মাটি (কাঁচা) .... 11<br>**প্রাথমিক পর্যায়ের দেয়াল ঃ**<br>কাঠ .... 21<br>**পরিপূর্ণ দেয়াল ঃ**<br>ইট/সিমেন্ট .... 31<br>টিন .... 32<br>অন্যান্য ________________________ 96<br>(নির্দিষ্ট কর ুন)	
1009	বসত ঘরের মেঝের প্রধান নির্মাণ-সামগ্রীঃ<br><br>**দেখে লিপিবদ্ধ কর ুন**	**কাঁচা মেঝে ঃ**<br>বাঁশ/মাটি (কাঁচা) .... 11<br>প্রাথমিক পর্যায়ের মেঝেঃ :<br>কাঠ .... 21<br>**পাকা মেঝে ঃ**<br>সিমেন্ট/ইট বালি জমানো .... 31<br>অন্যান্য ________________________ 96<br>(নির্দিষ্ট কর ুন)	
1010	আপনাদের খানার মালিকানায় বসত ভিটা আছে কি?<br>**যদি না হয়, প্রোব কর ুনঃ**<br>আপনাদের খানার অন্য কোথাও বসত ভিটা আছে কি?	হ্যাঁ ....1<br>না 2	
1011	আপনাদের (**খানার বসত ভিটা ছাড়া**) কোন জমি আছে কি?	হ্যাঁ ....1<br>না 2	→1013
1012	(**বসত ভিটা ছাড়া**) আপনার মালিকানায় কি পরিমাণ জমি আছে?<br>পরিমাণ: __________ **একক:** __________<br>(**1** কিয়ার = **30** শতাংশ বা ডেসিমেল)	☐☐ ☐☐<br>একর শতাংশ	
1013	আপনার খানায় (উত্তরদাতা নিজের সহ) কয়টি মোবাইল ফোন আছে?	সংখ্যা .... ☐☐<br>মোবাইল ফোন নাই 00	
1014	আপনার পরিবারের উপার্জনক্ষম সদস্য কতজন?<br>**(শুধুমাত্র খানার সদস্যদের সংখ্যা লিপিবদ্ধ কর ুন, বিদেশে অবস্থানরত খানা সদস্যদেরও অর্ন্তভূক্ত কর ুন)**	জন .... ☐☐	
1015	আপনার পরিবারের আনুমানিক মোট মাসিক আয় কত?	টাকা.............................. ☐☐☐☐☐	
1016	আপনার পরিবারের আনুমানিক মোট মাসিক খরচ কত?	টাকা.............................. ☐☐☐☐☐	

এখন আমি আপনাদের সংসারের গত ১ মাসের যাবতীয় খরচ সম্পর্কে কিছু প্রশ্ন জিজ্ঞেস করবো।	
1017	গত ১ মাসে আপনাদের সংসারে খাবার বাবদ কতটুকু খরচ হয়েছে বলুন। **কোন খরচ না হলে 0000 লিখুন।**

	দ্রব্যের নাম<br>**(প্রত্যেকটি সম্পর্কে জিজ্ঞেস করুন)**	A. ____ কতটুকু (দ্রব্য) ব্যবহৃত/খরচ হয়েছে?	B. সেটা কি নিজেদের চাষ করা, কেনা না-কি চাষ করা এবং কেনা উভয়ই?	C. কতটুকু বাজার থেকে কিনেছেন?	D. এই কেনা বাবদ মোট কত টাকা খরচ হয়েছে? (টাকায় লিখুন)
	a. চাল	□□□<br>কেজি	চাষ করা ....1<br>কেনা ....2<br>**(D এ যান)**<br>উভয়ই 3	□□<br>কেজি	□□□□
	b. আটা/গম	□□<br>কেজি<br>না 95	চাষ করা ....1<br>কেনা ....2<br>**(D এ যান)**<br>উভয়ই 3	□□<br>কেজি	□□□□
	c. চিনি	□□<br>কেজি<br>না 95			□□□□
	d. শস্যজাত দ্রব্য যেমন চিড়া, খই, মুড়ি, রুটি, ময়দা, নুডুলস, জোয়ার, বাজরা থেকে তৈরি দ্রব্য, কর্ণফ্লেক্স ইত্যাদি	ব্যবহৃত/খরচ হয়েছে.1<br>ব্যবহৃত/খরচ হয় নি..2	চাষ করা ....1<br>কেনা ....2<br>উভয়ই ....3<br>**(D এ যান)**		□□□□
	e. ডাল এবং ডাল থেকে তৈরি দ্রব্য যেমন সয়াবিন, বেসন ইত্যাদি	ব্যবহৃত/খরচ হয়েছে.1<br>ব্যবহৃত/খরচ হয় নি..2	চাষ করা ....1<br>কেনা ....2<br>উভয়ই ....3<br>**(D এ যান)**		□□□□
	f. মাংস, মুরগী, মাছ	ব্যবহৃত/খরচ হয়েছে.1<br>ব্যবহৃত/খরচ হয় নি..2	চাষ করা ....1<br>কেনা ....2<br>উভয়ই ....3<br>**(D এ যান)**		□□□□
	g. গুড় এবং অন্যান্য মিষ্টিদ্রব্য (মিছরি, মধু)	ব্যবহৃত/খরচ হয়েছে.1<br>ব্যবহৃত/খরচ হয় নি..2	চাষ করা ....1<br>কেনা ....2<br>উভয়ই ....3<br>**(D এ যান)**		□□□□
	h. সয়াবিন/সরিষার তেল	ব্যবহৃত/খরচ হয়েছে.1<br>ব্যবহৃত/খরচ হয় নি..2	চাষ করা ....1<br>কেনা ....2<br>উভয়ই ....3<br>**(D এ যান)**		□□□□
	i. ডিম<br>**১ হালির কম হলে 00 লিখুন।**	□□<br>হালি	চাষ করা ....1<br>কেনা ....2<br>উভয়ই ....3<br>**(D এ যান)**		□□□□
	j. দুধ / দুধজাত দ্রব্য যেমন ঘি, মাখন,	ব্যবহৃত/খরচ হয়েছে.1<br>ব্যবহৃত/খরচ হয় নি..2	চাষ করা ....1<br>কেনা ....2<br>উভয়ই ....3<br>**(D এ যান)**		□□□□
	k. বিভিন্ন ধরনের সবজি	ব্যবহৃত/খরচ হয়েছে.1<br>ব্যবহৃত/খরচ হয় নি..2	চাষ করা ....1<br>কেনা ....2<br>উভয়ই ....3<br>**(D এ যান)**		□□□□

	দ্রব্যের নাম (প্রত্যেকটি সম্পর্কে জিজ্ঞেস করুন)	A. ____ কতটুকু (দ্রব্য) ব্যবহৃত/খরচ হয়েছে?	B. সেটা কি নিজেদের চাষ করা, কেনা না-কি চাষ করা এবং কেনা উভয়ই?	C. কতটুকু বাজার থেকে কিনেছেন?	D. এই কেনা বাবদ মোট কত টাকা খরচ হয়েছে? (টাকায় লিখুন)
	l. লবন ও মশলা (হলুদ, গোলমরিচ, শুকনামরিচ, আদা, তেতুল, রসুন, সরিষা ইত্যাদিসহ)	ব্যবহৃত/খরচ হয়েছে.1<br>ব্যবহৃত/খরচ হয় নি..2	চাষ করা.................1<br>কেনা......................2<br>উভয়ই....................3<br>(D এ যান)		☐☐☐☐
	m. অন্যান্য খাবার যেমন চা, কফি, তৈরি খাবার যেমন বিস্কুট, কেক, আচার, সস ইত্যাদি	ব্যবহৃত/খরচ হয়েছে.1<br>ব্যবহৃত/খরচ হয় নি..2	চাষ করা.................1<br>কেনা......................2<br>উভয়ই....................3<br>(D এ যান)		☐☐☐☐
	n. ফলমূল (আম, কলা, নারকেল, খেজুর ও অন্যান্য শুকনো ফল)	ব্যবহৃত/খরচ হয়েছে.1<br>ব্যবহৃত/খরচ হয় নি..2	চাষ করা.................1<br>কেনা......................2<br>উভয়ই....................3<br>(D এ যান)		☐☐☐☐

1018	উপরের খাদ্য দ্রব্য এর জন্য গত এক মাসে আনুমানিক মোট কত টাকা খরচ হয়েছে? (উত্তর জানা না থাকলে বাড়ির অন্য কাউকে জিজ্ঞাসা করে জানতে বলুন)	টাকা ☐☐☐☐☐
1019	গত ১ মাসে _______ (বিষয়) বাবদ মোট কত টাকা খরচ হয়েছিল? **কোন খরচ না হলে 0000 লিখুন।**	(টাকায় লিখুন)
	a. জ্বালানী ও বিদ্যুৎ (জ্বালানী গ্যাস, বিদ্যুৎ, জ্বালানী কাঠ, কেরোসিন)	☐☐☐☐
	b. দৈনন্দিন ব্যবহার্য (টুথপেস্ট, তেল, সেভিং দ্রব্যাদি)	☐☐☐☐
	c. গৃহস্থালী দ্রব্য (বাল্ব, টিউব লাইট, বাসন, সাবান, বালতি ইত্যাদি)	☐☐☐☐
	d. বাড়ী ভাড়া/ভোক্তা কর ও বিল (পানির বিল সহ)	☐☐☐☐
	e. যাতায়াত খরচ (ট্রেন, বাস, ট্যাক্সি, রিকশা, পে- ন, ডিজেল, পেট্রোল, স্কুল ভ্যান ইত্যাদি)	☐☐☐☐
	f. ব্যক্তিগত সেবা (চশমা, ছাতা, টর্চ, লাইটার ইত্যাদি)	☐☐☐☐
	g. সেবা (গৃহকর্মীর বেতন, পারিশ্রমিক ইত্যাদি)	☐☐☐☐
	h. বিনোদন (সিনেমা, খেলাধুলা, পিকনিক, টেলিফোন, কেবল, ইন্টারনেট ইত্যাদি)	☐☐☐☐
	i. রেস্টুরেন্ট বা বাইরে খাবার খরচ	☐☐☐☐
	j. পান, তামাক, সিগারেট ইত্যাদি	☐☐☐☐
1020	উপরের যাবতীয় খরচের জন্য গত এক মাসে আনুমানিক মোট কত টাকা খরচ হয়েছে? (উত্তর জানা না থাকলে বাড়ির অন্য কাউকে জিজ্ঞাসা করে জানতে বলুন)	টাকা ☐☐☐☐☐

	**এখন আমি গত ১ বৎসরে আপনাদের সংসারে খাবার ছাড়া অন্যান্য খরচ কত হয়েছে সে সম্পর্কে জিজ্ঞেস করবো।**		
1021	গত ১২ মাসে _______ বাবদ কত টাকা খরচ হয়েছে? **কোন খরচ না হলে 0000 লিখুন।**<br>(বিষয়)	(টাকায় লিখুন)	
	a. স্কুল/প্রাইভেট টিউশন (প্রাইভেট টিউটর, স্কুল/কলেজ ফী)	☐☐☐☐☐	
	b. স্কুল বই ও অন্যান্য লেখাপড়ার উপকরণ	☐☐☐☐☐	
	c. জামা-কাপড়/শাড়ী/জুতা	☐☐☐☐☐	
	d. আসবাবপত্র ও অন্যান্য (বিছানার চাদর, আলমারী, সুটকেস, কার্পেট ইত্যাদি)	☐☐☐☐☐	
	e. তৈজসপত্র (থালাবাসন, সসপ্যান ইত্যাদি)	☐☐☐☐☐	
	f. রান্না ও গৃহস্থালী সামগ্রী (চুলা, কুকার, ওয়াশিং মেসিন, ফ্রিজ ইত্যাদি)	☐☐☐☐☐	
	g. বিনোদন সামগ্রী (টিভি, রেডিও, টেপ রেকর্ডার, মিউজিক সিস্টেম ইত্যাদি)	☐☐☐☐☐	
	h. গহনাগাটি	☐☐☐☐☐	
	i. ব্যক্তিগত যোগাযোগ (বাইসাইকেল, স্কুটার, গাড়ি, চাকা ইত্যাদি)	☐☐☐☐☐	
	j. চিকিৎসা দ্রব্যাদি (চশমা, কানে শোনার যন্ত্র ইত্যাদি)	☐☐☐☐☐	
	k. অন্যান্য ব্যক্তিগত দ্রব্য (ঘড়ি, কম্পিউটার, টেলিফোন, মোবাইল ইত্যাদি)	☐☐☐☐☐	
	l. মেরামত খরচ (বাড়িঘর মেরামত ইত্যাদি)	☐☐☐☐☐	
	m. ইন্সুরেন্স প্রিমিয়াম/কিস্তি	☐☐☐☐☐	
	n. বেড়ানো	☐☐☐☐☐	
	o. সামাজিক অনুষ্ঠান (বিয়ে, জন্মদিনের উপহার ইত্যাদি)	☐☐☐☐☐	
1022	মাসের সকল খরচাদি বাদে আপনাদের কোন সঞ্চয় হয় কি?	হ্যাঁ...1<br>না ....2<br>জানি না...7	2, 7 → 1024
1023	মাসে আনুমানিক কত টাকা সঞ্চয় হয়?	মোট সঞ্চয়.................... ☐☐☐☐☐ টাকা	
**1024**	**সাক্ষাৎকারগ্রহনকারীঃ প্রশ্ন 219 দেখুন এবং সঠিক কোড বৃত্তায়িত করুন।**	**0-29 দিন ....1**<br>**1 মাস বা তার অধিক..2**<br>**কোন উত্তর নেই 3**	**2, 3 → 1026**
**1025**	**সাক্ষাৎকারগ্রহনকারীঃ আপনার সুপারভাইজারকে জানান যে এই খানার বাচ্চা 0-29 দিন বয়সের মধ্যে মারা গিয়েছে।**		
**1026**	**সাক্ষাৎকারগ্রহনকারীঃ উত্তরদাতার কাছ থেকে বিদায় নেয়ার পূর্বে প্রশ্নমালাটি ভাল করে পরীক্ষা করে দেখুন। অতঃপর উত্তরদাতাকে ধন্যবাদ জানিয়ে সাক্ষাৎকার শেষ করুন।**		
